# The association of windmills with conservation of pollinating insects and wild plants in homogeneous farmland of western Poland

**DOI:** 10.1007/s11356-017-0864-7

**Published:** 2017-12-15

**Authors:** Sylwia Pustkowiak, Weronika Banaszak-Cibicka, Łukasz Emil Mielczarek, Piotr Tryjanowski, Piotr Skórka

**Affiliations:** 1 Institute of Nature Conservation, Polish Academy of Sciences, Mickiewicza 33, 31-120 Kraków, Poland; 2 Institute of Zoology, Poznań University of Life Sciences, Poznań, Poland; 3 Department of Pomology and Apiculture, Agricultural University in Kraków, Kraków, Poland

**Keywords:** Agricultural landscape, Biodiversity, Marginal habitat, Pollinator decline, Weed, Wind farm

## Abstract

Loss of suitable seminatural habitats and homogenization of crop types have led to the population decline of pollinating insects in farmland. As these insects support crop production, many practical efforts aim to sustain pollinator diversity which is especially challenging in intensively managed and homogeneous farmland. However, there are ongoing changes of the farmland toward its multifunctionality that includes, for example, wind farm development. Windmills are often built within crops; thus, we examined if the noncropped area around windmills can be valuable habitats for wild plants and pollinating insects: bees, butterflies, and flies. Species richness, abundances, and species diversity index of plants and pollinators around windmills were similar to those found in grassland patches (a typical habitat for these insects) and higher than in the adjacent crops. Pollinator diversity index and species richness at windmills increased with the distance to the nearest grassland patch and windmill. The population sizes of pollinating insects were also positively associated with plant diversity. Particular groups of pollinators showed specific habitat associations: bees occurred mostly at windmills, butterflies were highly associated with grasslands, while flies occurred in a similar number at windmill and on grasslands. Since windmills are frequently built within extensive homogeneous fields, thus, they introduce pollination services into the interior of cropped areas, contrary to field margins, road verges, or seminatural grasslands. Thus, although the development of wind farms has various negative environmental consequences, they can be alleviated by the increase of the local population size and diversity of wild plants and pollinating insects at windmills.

**Electronic supplementary material:**

The online version of this article (10.1007/s11356-017-0864-7) contains supplementary material, which is available to authorized users.

## Introduction

Farmland is an important habitat for many taxa (Pimentel et al. 1992; Söderström et al. 2001; Scherr and McNeely 2008; Rosin et al. 2016a). However, the intensification of farming has led to habitat fragmentation, habitat loss, and pollution of farmland and, in a consequence, to collapse or diminishing of ecosystem services (Green et al. 2005; Stoate et al. 2009). One of the most threatened ecosystem service is pollination of plants, which plays a key role in the food production and sustaining wild plant species diversity (Potts et al. 2010; Sekercioglu et al. 2010; Baude et al. 2016). The estimated number of pollinator species reaches over 1200 for vertebrates and nearly 300,000 for invertebrates which are responsible for pollination of over 90% of flowering angiosperms and 95% of crop plants (Nabhan and Buchmann 1997). The most recognized groups of pollinators are bees, but there are also other taxa such as flies, butterflies, birds, or bats that may contribute to pollination substantially (Rader et al. 2016).

The literature highlights a number of factors responsible for the disappearance of pollinators, such as the use of pesticides, mechanical cultivation, parasites infection, unavailability of food plants, invasive plant species, or climate change (Kevan and Viana 2003; Moroń et al. 2009; Lenda et al. 2010), many of which are highly related to intensive agriculture practices. The most frequently mentioned factor is fragmentation and loss of habitats, mainly seminatural grasslands with evidences that rising distance to those habitats in homogeneous farmland causes disturbances in plant-pollinator interactions and reduces the production of seeds and fruits (Ricketts et al. 2008; Jakobsson and Ågren 2014). Habitat loss and fragmentation also negatively affect food resources for pollinators, which are flowering arable weeds. This group of plants may be also breeding sites and larval host plants for these insects. Weeds are vanishing in intensively managed agricultural landscapes (Andreasen et al. 1996; Hyvönen et al. 2003; Chamorro et al. 2016) that homogenize and simplify plant-pollination network, speeding up the decline of diversity of pollinating insects (Baude et al. 2016).

Agricultural landscapes play numerous functions for humans apart from food production. For example, in fields, there may be built windmills, solar panels, silos, and other structures. These man-made structures are frequently described as negatively affecting local biota, but surprisingly, some may also attract or even play an important role for numerous species (e.g., electricity pylons, see: Tryjanowski et al. 2013; Rosin et al. 2016a). These marginal habitats, often regarded as “novel ecosystems” (Hobbs et al. 2009; Tropek et al. 2013; Moroń et al. 2014), are a unique combination of environmental features that do not usually exist in nature. Such specific conditions may cause the formation of new species assemblages (Lundholm and Richardson 2010; Lenda et al. 2012). There is growing evidence that highly transformed and degraded patches, like quarries, railway embankments, or road verges, can paradoxically become a surrogate habitat for many species including those of high conservation status, when their natural habitats disappear in agricultural landscapes (Beneš et al. 2003; Heneberg et al. 2012; Lenda et al. 2012; Moroń et al. 2014, 2017; Berg et al. 2016).

Increasingly popular anthropogenic elements in farmland are wind farms which produce carbon-free energy although their positive effect on the environment is controversial (Rosin et al. 2016b). For example, in Poland, the first windmill was built in 1991; in 2011, the installed power was 1800 MW, but at the end of 2015, the power grew to 4886 MW (Central Statistical Office of Poland 2016). Now in Poland, there are 1193 windmills with a total capacity 5807 MW.^1^ The most discussed issue on wind farm is bird and bat collisions with turbines (Rydell et al. 2010; Rosin et al. 2016b). There are also other problems like noise generated by turbines and alteration to the microclimate of adjacent fields including increased temperature of the ground, modification of humidity of the air at ground level, and heat transfer between the ground and atmosphere (Roy 2011; Walsh-Thomas et al. 2012).

However, windmills may also benefit some of the farmland species as they provide new microhabitats and specific vegetation in the homogenized landscape dominated by crops. Around the windmill, there is a part of unused space spontaneously overgrown by flowering weeds (Supplementary material 1, Figs. S1 and S2). This suggests that windmills may provide surrogate habitats for some wild species including plants and pollinating insects. The aim of this study is to assess the role of windmill site in enhancing biodiversity of weeds and pollinating insects in a homogeneous landscape. We compared species composition in three habitats: areas around windmills (called simply “windmills” throughout the paper), in the grassland patches (typical habitat for pollinating insects; Abrol 2012), and in adjacent arable fields to assess potential contribution of each habitat to species richness, abundance, and diversity of weeds and pollinating insects. To our knowledge, there is currently no study analyzing the importance of windmill structures for sustaining biodiversity.

## Materials and methods

### Study area

The study was conducted from May to August 2014 at a wind farm in Gołańcz (52° 56′ 38″ N, 17° 17′ 58″ E) in a typical homogeneous agricultural landscape of western Poland. The whole district is covered in about 76% by arable land, while forests occupy only 15% of the district area (Central Statistical Office of Poland 2013).

This wind farm consists of 53 windmills, each with nominal power of 1.5 MW and height equal up to 120 m.^2^ Windmills are loosely dispersed on arable fields within an area of 48.5 km^2^. The construction of windmills involves the transformation of relief including the elimination of existing form of land use. Usually at each windmill, there is a graveled access square with approximate dimensions 20 × 30 m used by power station service (Supplementary material 1, Figs. S1 and S2). The edge of this square and the area around the tower of windmill are overgrown by segetal vegetation. The studied landscape includes also seminatural grasslands, mainly *Arrhenatheretum elatioris* mesotrophic communities or purple moorgrass meadow *Molinietalia caeruleae* (Supplementary material 1, Fig. S3) with patchy distribution and single or double mowing during vegetation period.

**Fig. 1:**
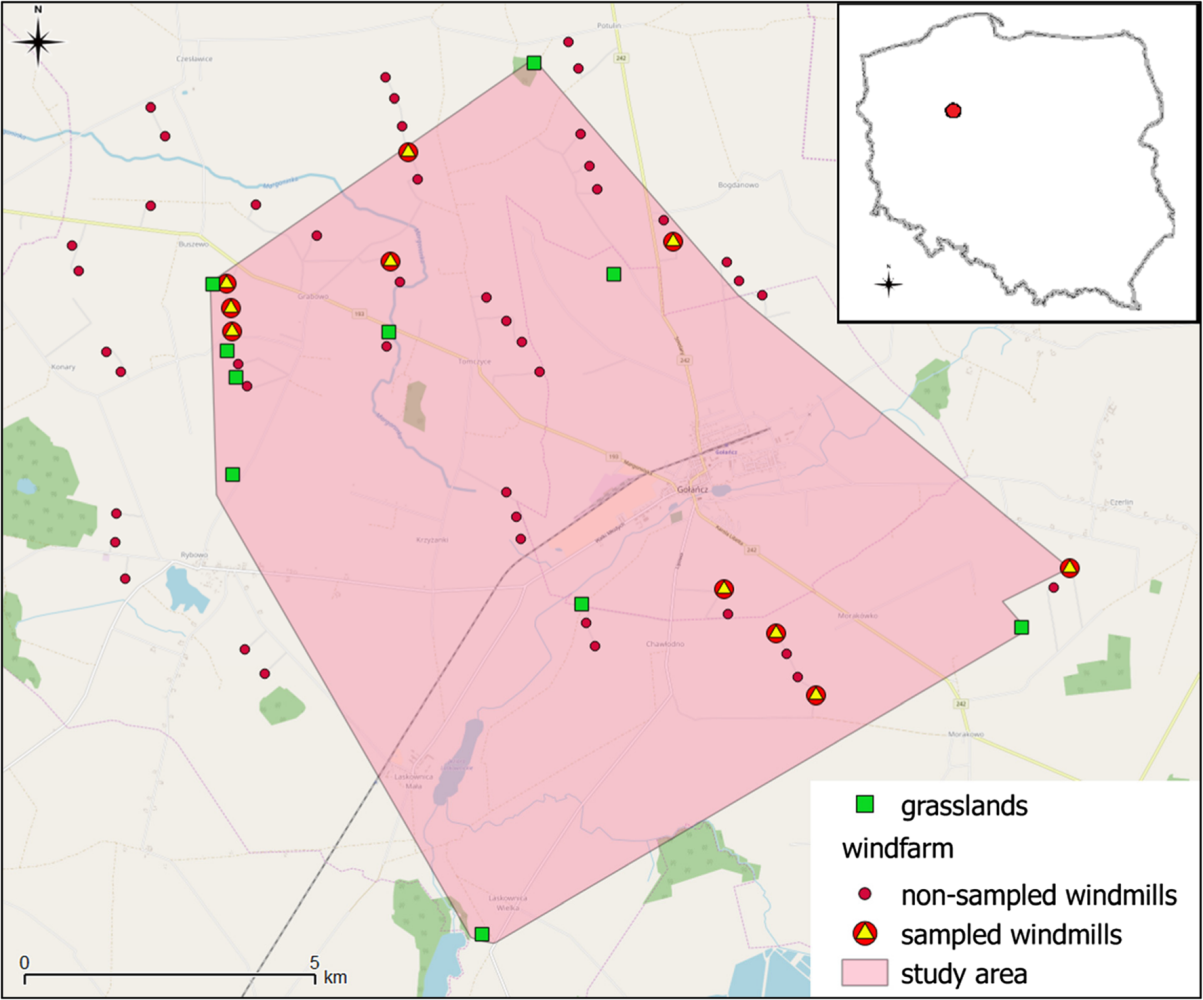
A map of the study area with the distribution of windmills (both sampled and nonsampled) and location of grassland patches

We selected 10 windmills and 10 control grasslands as study sites (Fig. 1). Each site was surrounded by field crops (14 cereal crops and 6 rape fields). Cereal and rape fields were equally divided between windmills and grasslands. Since there were no differences in pollinator diversity and abundance between two types of crops (pollinators *H*′ diversity index: generalized linear mixed model (GLMM) *F*_1, 35_ = 0.064, *p* = 0.802; total pollinators species GLMM *F*_1, 37_ = 0.021, *p* = 0.885; total pollinators abundance GLMM *F*_1, 37_ = 0.646, *p* = 0.421) nor did we find differences in pollinators diversity and abundance between fields adjacent to windmill and fields bordering with grasslands (pollinators *H*′ diversity index: *t*_16.74_ = − 0.596, *p* = 0.559; total pollinators species: *t*_17.32_ = 0.062, *p* = 0.952; total pollinators abundance: *t*_14.72_ = − 0.663, *p* = 0.518), we treated these fields as one habitat type hereafter referred to as “field” (see “Data analysis and statistics”). Control grasslands were small patches of a mean size (± SD) equalling 1.0 ± 0.87 ha (min = 0.14 ha; max = 2.76 ha), separated from each other by an average distance of 2021 ± 1493 m (min = 285 m; max = 4575 m). Sampled windmills were separated from each other by 1103 ± 1014 m (min = 244 m; max = 2942 m). The mean distance between two closest plots (windmill or grassland) was 835 ± 765 m (min = 154 m; max = 3579 m).

Fieldworks were conducted during clear weather with low wind speeds (up to 3 on the Beaufort scale), minimum air temperature of 17 °C, and cloud cover up to 30%. Windmills occasionally operated during surveys depending on the wind power. Insects were caught between 9:00 a.m. and 4 p.m. during three surveys (first—May/June [rape flowering period]; second—early July; third—end of July). At each windmill, we marked four sampling points: one in the center of the square and three near its edge between the square and the field (Fig. S1 in Supplementary material 1). Four consecutive points were located along a transect in the field every 15 m from the square edge into the field (Fig. S1 in Supplementary material 1). Within the grassland patches, we have distributed four points in a similar way to windmills encompassing the area of similar size as the windmill site and determining the next four points along a field transect. In total, within a study area, we had 160 sampling points, and on each of them there were three counts, giving a total of 480 samples (Fig. S1 in Supplementary material 1). The insects were caught in an entomological net, and each count consisted of 50 sweeps of net in a 2-m radius (approximate sampling time 1 min). Threatened species of bumblebees were determined in place and released. The study material was kept in 70% ethanol until the taxonomic revision. The insects (bees, butterflies, and flies) were pinned and then determined according to species. Within each sampling point, we recorded data on vegetation in a radius of 2 m, including the total percentage cover and the list of plant species with percentage cover of each species.

Due to generally the small number of insects, we pooled data from sampling points within each site (for each windmill, grassland, and field).

### Data analysis and statistics

To evaluate differences in plant and pollinator species richness, abundance, and Shannon *H*′ diversity index between habitats, we used GLMMs with Poisson error and log-link function (or Gaussian error distribution and identity link in case of the diversity index and plant cover) built in R version 3.3.2 (R Core Team 2016) using the “lme4” package (Bates et al. 2015). The fixed categorical variable was habitat type (three levels: windmill, grassland, field). As a random factor, we included a site identity (a pair windmill-field or grassland-field). We identified statistically significant differences between levels of the habitat type via paired contrasts in GLMMs (Quinn and Keough 2002). In each model, we included two covariates: the distance to the nearest grassland patch (DistanceG) and distance to the nearest windmill (DistanceW) to control for the effect of spatial arrangement of these habitats in a landscape. We also allowed the interaction terms between habitat type (three levels: windmill, grassland, field) and DistanceG as well as habitat type and DistanceW to control for possibly different responses of species groups to the proximity of grasslands and windmills in different habitat types. However, if any interaction term was statistically nonsignificant, it was removed from a final model (Bolker et al. 2008). DistanceG and DistanceW were log-transformed to avoid impact of detached observations (Quinn and Keough 2002). We did these analyses separately for bees (in two ways: with and without honeybee), butterflies, and flies. We also checked if the spatial autocorrelation affected the results of the generalized linear mixed models by calculating Moran I statistics for models’ residuals (Dormann et al. 2007) in R package “pgrimes” (Giraudoux 2017). However, there was no indication of spatial autocorrelation in the dependent variables (Fig. S4 in Supplementary material 1). To analyze how windmills influence species composition of pollinators and plants in a landscape, we conducted canonical correspondence analysis (CCA) with plot identity as supplementary variable using Canoco 5 (Lepš and Šmilauer 2003). We estimated an impact of each explanatory variable to ordination by calculating pseudo-*F* statistics with *p* values given after the Bonferroni correction. This analysis was also conducted separately for the three pollinator groups studied. Co-correspondence analysis (CoCa) was used to test if plant species composition in windmills correlate with species composition of all pollinating insects (ter Braak and Schaffers 2004).

## Results

In the study period, we captured a total of 299 individuals including 137 flies, 87 butterflies, and 75 bees (Table 1). The most abundant species was honeybee (15% of total material, 60% of all bees). The most species rich group was *Diptera* order, represented mainly by *Syrphidae* family; 70% of all captured butterflies belonged to *Pieridae* family. Three hundred and twenty-five of the 480 samples contained no pollinator. In one site (a field), we did not catch any pollinating insect. Within all study plots, we noted 134 plant species. The list of all pollinators and plant species is available in Supplementary material 1 (Tables S1 and S2).

**Table 1:** List of taxons and abundances of captured pollinators (the sum of the three controls)

Taxon (family)	Number of species	Abundance
*Colletidae*	1	2
*Andrenidae*	2	3
*Halictidae*	3	6
*Megachilidae*	1	1
*Apidae*	8	63
Total *Hymenoptera*	15	75
*Hesperiidae*	2	8
*Lycaenidae*	1	1
*Nymphalidae*	6	17
*Pieridae*	3	61
Total *Lepidoptera*	12	87
*Anthomyiidae*	1	1
*Stratiomyidae*	3	14
*Syrphidae*	19	121
*Tachinidae*	1	1
Total *Diptera*	24	137

### Effect of windmills on total numbers of pollinators

**Fig. 2:**
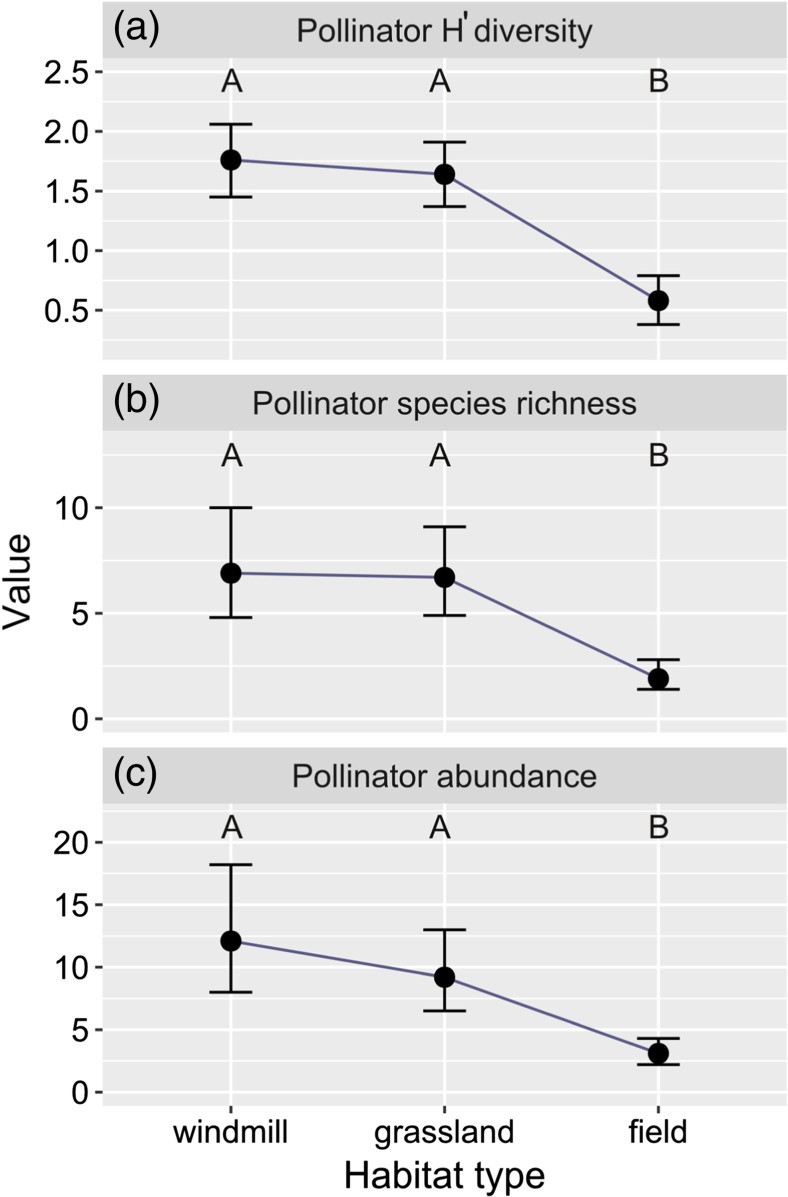
The relationship between habitat type and pollinator Shannon diversity *H*′ index (**a**), total number of pollinators species per plot (**b**), and pollinator abundance within a plot (**c**). Points represent means estimated in generalized linear mixed models. Error bars show 0.95% confidence level also derived from generalized linear mixed models

**Fig. 3:**
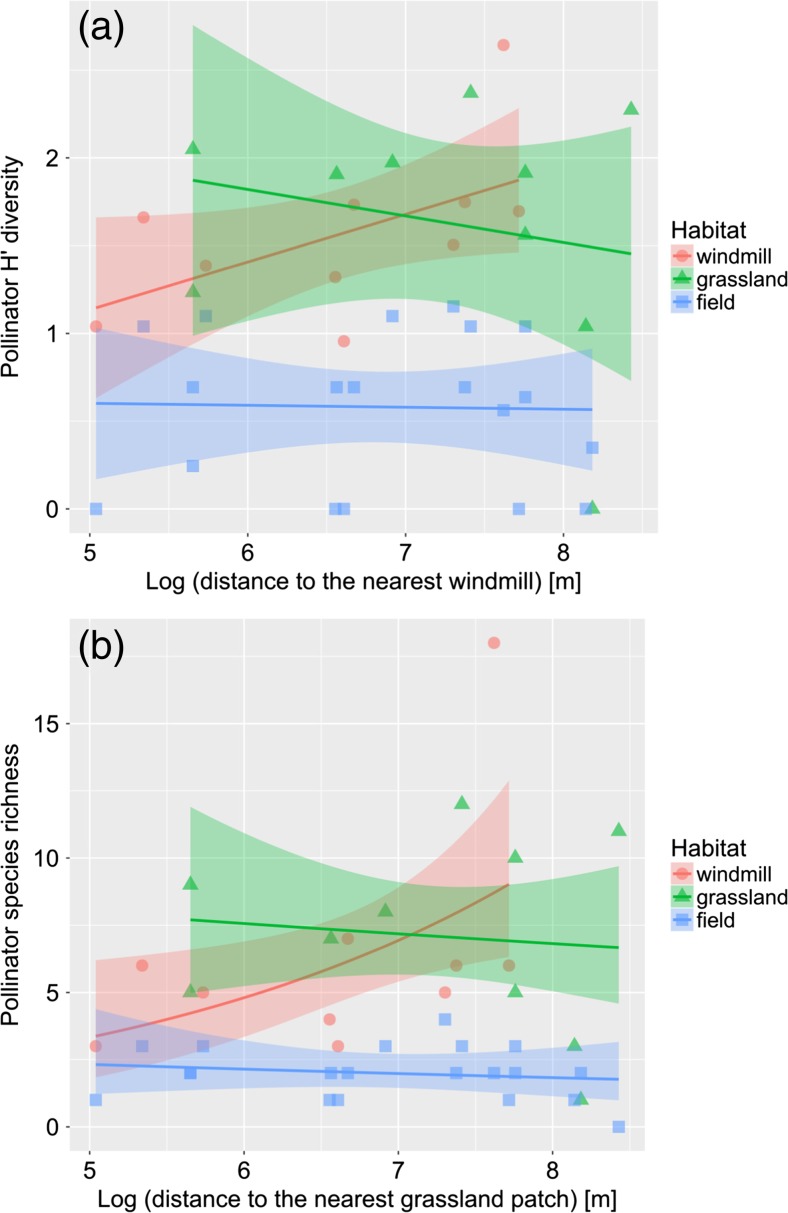
The effect of distance to the nearest windmill (**a**) and grassland patch (**b**) on pollinator Shannon diversity index and species richness, respectively. Shaded bands represent 95% confidence intervals

The studied habitats significantly differ in pollinator diversity, species richness, and abundance (pollinators *H*′ diversity index GLMM1 *F*_2, 34_ = 33.32, *p* < 0.001; total pollinators species GLMM2 *F*_2, 35_ = 22.39, *p* < 0.001; total pollinators abundance GLMM3 *F*_2, 36_ = 37.59, *p* < 0.001). For all those three response variables, fields have lower values than windmill sites and grasslands (Table 2). However, there were no significant differences in pollinators diversity and abundance between windmill sites and grasslands (Table 2, Fig. 2a–c). Species diversity index at windmills (but not in grassland patches or fields) increased with the distance to the nearest windmill (Table 2, Fig. 3a). Pollinator species richness increased with the distance to the nearest grassland patch (Table 2, Fig. 3b).

**Table 2:** Summary of generalized linear mixed models explaining diversity, species richness, and abundance of pollinators in the three studied habitats. Windmill site is used as a reference level of habitat. Plot ID is used as a random factor. Explanations: DistanceW—distance to the nearest windmill, DistanceG—distance to the nearest grassland. Significant effects are marked in bold

Explanatory variables	Estimate	SE	*t*/*z* value	*p* value
GLMM1 (*H*′ diversity index)				
Intercept	1.75	0.16	11.13	**< 0.001**
Habitat = grassland	− 0.08	0.19	− 0.39	0.700
Habitat = field	− 1.14	0.15	− 7.39	**< 0.001**
DistanceW	0.03	0.15	0.20	0.841
DistanceG	0.12	0.11	1.05	0.305
Habitat = grassland * DistanceW	− 0.56	0.18	− 3.04	**0.006**
Habitat = field * DistanceW	− 0.15	0.13	− 1.94	0.247
Habitat = windmill	0*			
GLMM2 (species richness)				
Intercept	1.94	0.15	12.73	**< 0.001**
Habitat = grassland	− 0.06	0.22	− 0.29	0.772
Habitat = field	− 1.29	0.21	− 5.97	**< 0.001**
DistanceW	− 0.17	0.11	− 1.48	0.139
DistanceG	0.42	0.17	2.43	**0.015**
Habitat = grassland * DistanceG	− 0.41	0.21	− 1.88	0.060
Habitat = field * DistanceG	− 0.45	0.22	− 2.01	**0.044**
Habitat = windmill	0*			
GLMM3 (abundance)				
Intercept	2.45	0.16	15.50	**< 0.001**
Habitat = grassland	− 0.21	0.21	− 1.00	0.325
Habitat = field	− 1.30	0.18	− 7.23	**< 0.001**
DistanceW	− 0.12	0.13	− 0.99	0.321
DistanceG	0.12	0.13	0.89	0.371
Habitat = windmill	0*			

*A reference category

**Fig. 4:**
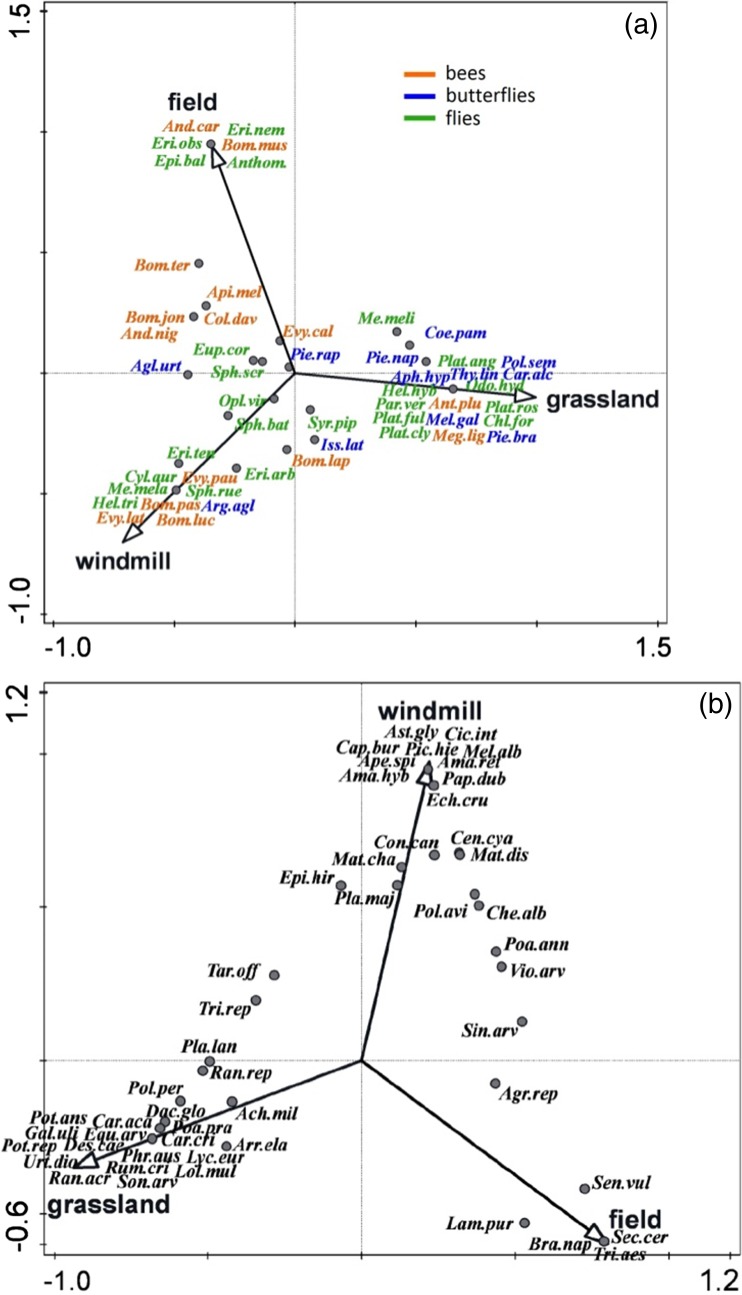
The CCA diagrams of relationships between habitats and **a** pollinators and **b** plants along the first and second ordination axis. Species abbreviations are explained in Tables S1 and S2 in Supplementary material 1

Canonical correspondence analysis revealed that there were significant differences in pollinator species composition among habitat types (test of first ordination axis: pseudo-*F* = 2.5, *p* = 0.002; test of all axes: pseudo-*F* = 1.9, *p* = 0.002). The first two axes explained 9.5% of variation in species composition. Variables that statistically contributed to the ordination were windmill sites (pseudo-*F* = 1.9, *p*_adj._ = 0.006) and grasslands (pseudo-F = 2.6, *p*_adj._ = 0.006) which accounted for 4.8 and 6.5% of the variation in the pollinator species data, respectively (Fig. 4a).

### Effect of windmills on plants

**Fig. 5:**
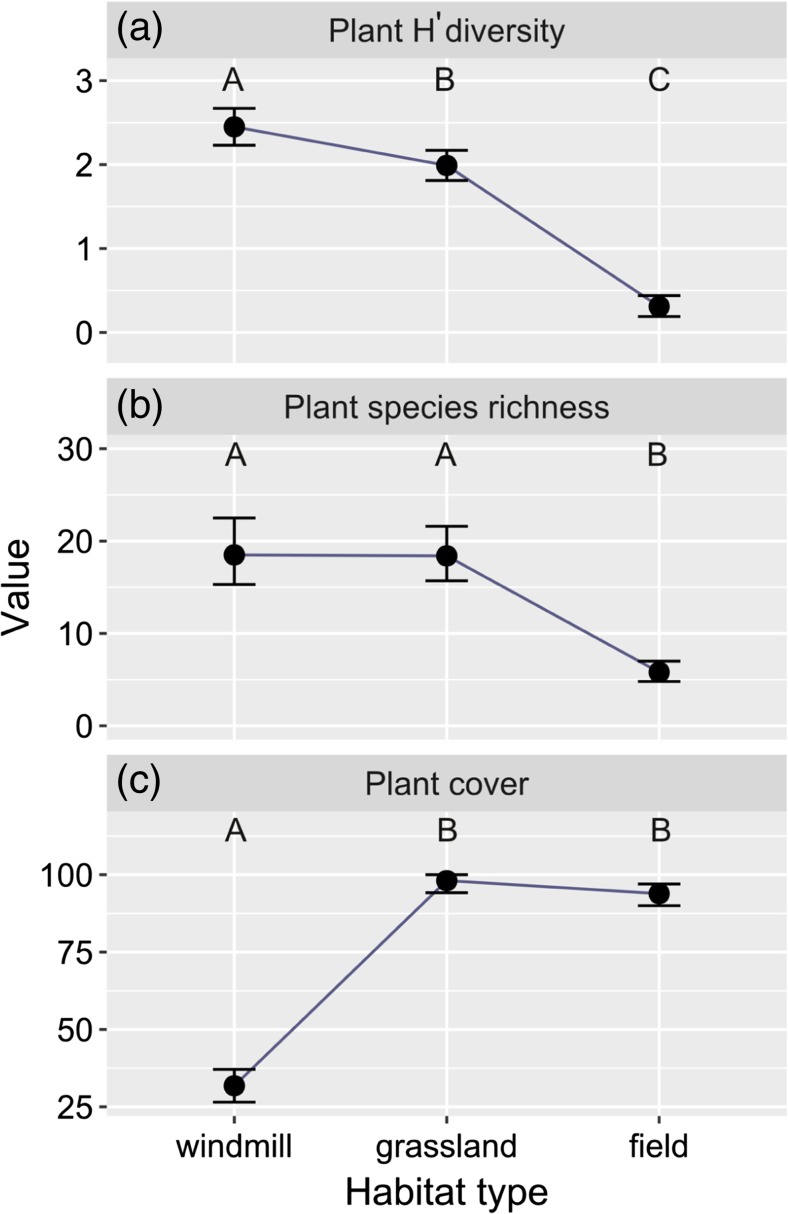
The relationship between habitat type and plant Shannon diversity *H*′ index (**a**), total number of plant species (**b**), and mean plant cover within plots (**c**). Points represent means estimated in generalized linear mixed models. Error bars show 0.95% confidence level also derived from generalized linear mixed models

There were differences in plant species *H*′ diversity index, species richness, and cover among habitats (plants *H*′ diversity index GLMM4 *F*_2, 35_ = 268.62, *p* < 0.001; total plants species GLMM5 *F*_2, 36_ = 61.545, *p* < 0.001; plant cover GLMM6 *F*_2, 35_ = 370.2, *p* < 0.001). Plants *H*′ diversity index was lower on grasslands and fields than at windmills (Table 3, Fig. 5a). The total number of plant species was lower on fields than on windmill sites and grasslands, while plant cover was significantly higher on grasslands and fields than on windmill sites (Table 3, Fig. 5b, c).

**Table 3:** Summary of generalized linear mixed models explaining diversity index, species richness, and cover of plants in the three studied habitats. Windmills are used as a reference level for habitat variable. Plot ID is used as a random factor. Explanations: DistanceW—distance to the nearest windmill, DistanceG—distance to the nearest grassland. Significant effects are marked in bold

Explanatory variables	Estimate	SE	*t*/*z* value	*p* value
GLMM4 (*H*′ diversity index)				
Intercept	2.44	0.09	27.97	**< 0.001**
Habitat: grassland	− 0.45	0.13	− 3.3	**0.002**
Habitat: field	− 2.12	0.11	− 20.04	**< 0.001**
DistanceW	0.01	0.06	0.184	0.855
DistanceG	− 0.04	0.05	− 0.68	0.502
Habitat: windmill	0*			
GLMM5 (species richness)				
Intercept	2.94	0.07	39.38	**< 0.001**
Habitat: grassland	− 0.03	0.11	− 0.25	0.804
Habitat: field	− 1.19	0.12	− 10.01	**< 0.001**
DistanceW	0.04	0.06	0.66	0.510
DistanceG	− 0.08	0.06	− 1.19	0.232
Habitat: windmill	0*			
GLMM6 (plant cover)				
Intercept	33.29	2.08	16.01	**< 0.001**
Habitat: grassland	65.20	3.00	21.76	**< 0.001**
Habitat: field	60.66	2.48	24.48	**< 0.001**
DistanceW	− 0.75	1.39	− 0.54	0.598
DistanceG	0.475	1.42	0.34	0.741
Habitat: windmill	0*			

*A reference category

The CCA performed for plant communities revealed that they were also significantly different among habitat types (test of the first axis pseudo-*F* = 3.4, *p* = 0.002; test of all axes pseudo-*F* = 3.1, *p* = 0.002). All three types of habitat had significantly different plants species (windmills pseudo-*F* = 2.4, *p*_adj._ = 0.006; grasslands pseudo-*F* = 3.4, *p*_adj._ = 0.006; fields pseudo-*F* = 3.1, *p*_adj._ = 0.006). Windmill sites and grasslands explained 5.8 and 8.1% of variation in plant species composition, respectively, while fields explained 7.5% of variation (Fig. 4b). The first two CCA axes explained 14.2% of variation in species composition.

**Fig. 6:**
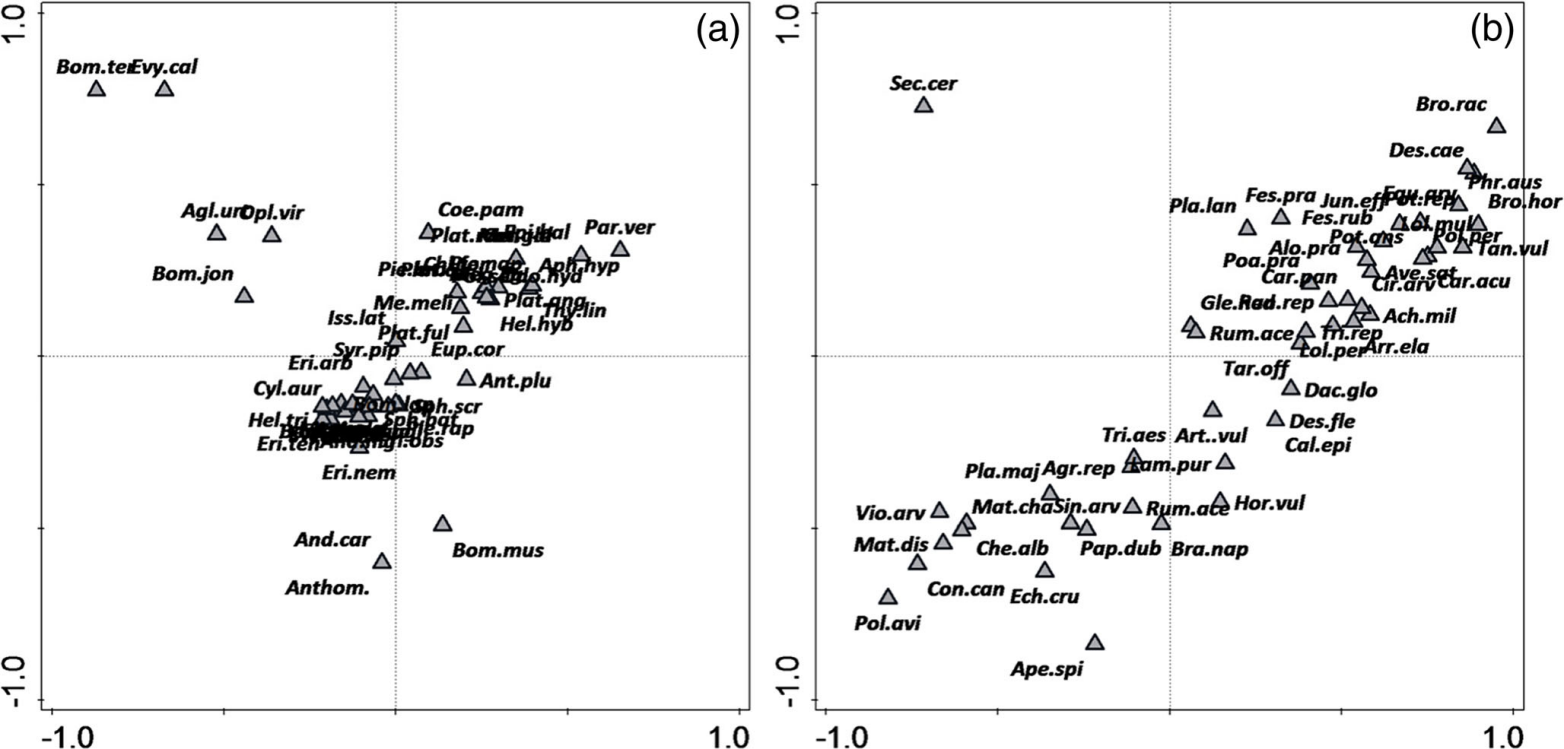
Biplot based on the symmetric co-correspondence analysis of the pollinator community (**a**) and plant community (**b**) within the studied area

Finally, co-correspondence analysis was performed to assess the covariance between pollinator and plant communities. The first two axes of CoCa analysis explained 33.8% of variation in the pollinators and plants data. The test of all axes showed a significant relation between pollinator and plant assemblages (trace = 3.71, *p* = 0.04, Fig. 6a, b).

### Individual pollinator group response to windmills

Habitat associations for particular groups of pollinators were described by GLMMs summarized in Table S3 in Supplementary material 1. Bees had the highest *H*′ diversity index, species richness, and abundance at windmills, while grassland and fields had significantly less species and individuals (Fig. S5a–c in Supplementary material 1**)**. After removing the honeybee from the analysis, windmills had higher species diversity index and species richness than grasslands and fields (Table S3, Fig. S5d, e). The number of wild bee individuals at windmills was higher than in fields but not statistically different from grasslands (Table S3, Fig. S5f in Supplementary material 1). Bees also responded to the proximity of other grassland patches and windmills (Table S3). Bee species richness (with or without honeybee) and wild bee abundance increased with the distance to the nearest grassland patch (Table S3, Fig. S6a–c in Supplementary material 1). Moreover, wild bee species diversity index at windmills (but not grasslands) decreased with distance to the nearest windmill (Table S3, Fig. S6d).

For butterflies, there were significantly higher species *H*′ diversity index, species richness, and abundance within grasslands than at windmills and in fields; however, windmills had substantially more butterfly individuals than fields (Fig. S7 in Supplementary material 1). Butterfly abundance decreased with the distance to the nearest windmill regardless of habitat type (Table S3).

For flies, the effect of windmills was similar as for all pollinators pooled: fields had a lower *H*′ diversity index, species richness, and abundance than windmills or grasslands and with no differences between the latter two (Fig. S8 in Supplementary material 1).

Species composition of particular pollinator groups was related to habitat type in butterflies (test of first axis pseudo-*F* = 2.9, *p* = 0.002; test of all axes pseudo-*F* = 1.6, *p* = 0.018) and flies (test of first axis pseudo-*F* = 2.1, *p* = 0.002; test of all axes pseudo-*F* = 1.7, *p* = 0.002, Fig. S9b, c), but not in bees (test on first axis pseudo-*F* = 1.5, *p* = 0.242; test on all axes pseudo-*F* = 1.1, *p* = 0.294, Fig. S9a). The exclusion of the honeybee from the analysis did not alter the results of the ordination for bees (test on first axis pseudo-*F* = 1.2, *p* = 0.362; test on all axes pseudo-*F* = 1.1, *p* = 0.284, Fig. S9d). The first two CCA axes explained 12.8% of variation in the composition of butterflies composition and 11.2% of the composition of flies. Grasslands and windmill sites contributed to community differentiation in butterflies and flies, but not in bees. Detailed tests on the effects of the explanatory variables are summarized in Table S4 in Supplementary material 1.

## Discussion

### Habitats for pollinators and plants

We demonstrated that windmill sites supported equal species richness, diversity, and abundance of pollinating insects as their typical habitat—seminatural grassland patches and higher than species-poor crop fields. Moreover, species composition was different in windmills, grasslands, and fields, indicating that windmills contribute to species diversity at the landscape scale in the study area. Not only populations of pollinating insects were supported by windmills. Weed species richness and diversity was the highest at windmills. As in pollinators, each of the three habitat types was characterized by a different plant species composition. These results have important implications for the functioning and conservation of plant-pollinator network in a homogeneous farmland.

Marginal habitats (some of them being also novel ecosystems) such as balks, fallows, ditches, road verges, and field borders may enable maintaining species diversity when there is a lack of natural habitats (Jankowiak and Ławicki 2014; Moroń et al. 2014; Piekarska-Boniecka et al. 2015; Assandri et al. 2016). Many analyses paid attention to the role of these marginal habitats in spatial dynamics of insects and insect-pollinated plants (Banaszak 1992; Raemakers et al. 2001; Ricketts et al. 2008; Jakobsson and Ågren 2014). In homogeneous landscapes, these marginal areas may be a habitat surrogate crucial for the survival of populations of endangered species. Such areas function often as movement and dispersal corridors or stepping stones in inhospitable matrix as pollinators rely on food and nesting sources that have patchy distribution (Kajzer-Bonk et al. 2016; Moroń et al. 2017). The positive effect of proximity of other windmills on wild bee diversity index and butterfly abundance indicates that at least for some species windmills may increase population connectivity in a landscape. Nevertheless, it would be interesting to investigate the influence of windmill network on pollinator movements and pollen propagation across the landscape. What is unique for windmills is that they are predominantly arranged in rows and connected with roads, thus may act as stepping stones enhancing permeability of a landscape for pollinators.

High pollinator diversity and species richness even at isolated windmills indicates that these sites may act as a biodiversity hot spot inside extensive fields. It remains unclear if these marginal habitats may exist as independent and self-sufficient units as there are studies showing that the amount of supported biodiversity depends on the proximity of seminatural grassland habitats that function as a population source (Öckinger and Smith 2007; Jauker et al. 2009; Lenda and Skórka 2010). However, in our study area, windmills that were more distant from grasslands had more pollinating species (especially bees) and species composition at windmills was different from that in grassland plots. This suggests that windmills may be independent habitat patches able to sustain local populations and specific species communities. Another plausible explanation of different pollinator species composition among habitats is species filtering at early stage of succession (Lebrija-Trejos et al. 2010).

In forming species-rich communities of pollinating insects, often human disturbance and spontaneous succession of early stage vegetation play a role. Evidences show that the growing vegetation provides a source of food, but with the progress of succession, the availability of bare ground becomes limited (Tropek et al. 2010, 2016). This is the reason why sometimes conservation approaches involving the ban of any management lead to vegetation overgrowth and disappearance of some ground-nesting species. In this way, many natural reserves in central Europe which used to comprise high diversity of plants, bees, and wasps in the 1940s and 1950s now are lacking several ground-dwelling species of pollinators (Kosior et al. 2007; Tropek et al. 2010). The same situation applies to reclamation of postindustrial areas (Heneberg et al. 2012). A limited percentage cover of vegetation around windmills may also be a factor supporting the colonization of these areas by bees since it is known that some ground-nesting species prefer places with bare ground (Cane 1991). Other features, like the presence of stony ground, slopes, and heaps of excavated soil under a windmill, might also be important.

The high value of plant diversity *H*′ index at windmills indicates that these plots are characterized by a large variety of species and equability of cover of each species while grasslands are rather dominated by grass species with lower cover of flowering dicotyledons. The pollinators are dependent on plants, including weeds, and the stability of pollination networks and services requires the simultaneous protection of floral diversity and pollinators (Rollin et al. 2016). In our study plots, we examined plants as potential food base, but little is known about nesting opportunities for these insects provided by plants. The strong dependence of the pollinator community on plant communities revealed by CoCA analysis suggests that both food and nesting sites may play a role. Moreover, windmills may be a safer place for nesting than grasslands and fields due to limited management (mowing was made once about mid-August in studied windmills). However, there are several threats to plants and pollinators at windmills such as spraying with herbicides and pesticides (a common practice on the graveled windmill squares) or some maintenance earthworks, e.g., melioration. Therefore, future research in the wind turbine landscapes should focus on estimation nest site availability, nest site preferences and reproduction of selected species, and alleviation of potential threats.

Despite that we demonstrated a general positive impact of windmills on pollinator diversity, there were differences in responses between taxonomical groups of pollinators. Butterflies were the most specialized pollinators that were found to inhabit mostly grasslands, probably because grasses with flowering plants may support many species at different life stages (Settele et al. 2009). There was an interesting result for bees as they were significantly more diverse and abundant around a wind turbine, but their communities did not differ significantly between the analyzed habitats. The analysis conducted with the exclusion of domesticated honeybees also suggested the superior value of the windmill area for wild bees. Social bees in particular require appropriate nesting site with proximity of floral sources to feed the larvae and these two needs are best met in habitats around the turbine. In turn, the semimoist grasslands in our study site are more likely to be used by hygrophilous species of flies than by xerophilous bees (Bańkowska 1980). Although different species have different habitats and food requirements at various life stages, some species were closely associated with windmill sites and we can state that these structures participate in increasing the diversity of these groups at the local and landscape scales.

As bees and butterflies responded in different ways to windmills, flies turn out to be the most widely spread group of insects. Adult syrphids are often mentioned as important food-specialized pollinators (Ssymank et al. 2008; Biesmeijer et al. 2006), but they are given less attention in the literature (Larson et al. 2001). As other pollinators such as honeybees or bumblebees cannot effectively support pollination in all crops and wild plants alone (Potts et al. 2010), therefore, sustaining a high diversity of plant species composition requires participation of both generalist and specialist pollinators (Fontaine et al. 2005). Thus, the role of flies in maintaining plant-pollinator network should be investigated more thoroughly in future studies.

There are also other groups of animals that could potentially use windmill sites (Dudek et al. 2015). For example, small mammals were found by Łopucki and Mróz (2016) to occupy wind farms with similar abundance and species composition as control sites. However, there are also groups of animals such as birds that may be highly negatively affected by windmills (Rosin et al. 2016b); thus, the potential value of windmills for sustaining local species diversity should also consider different responses of different taxonomic groups to windmills.

### Practical recommendations

The ability of pollinators and plants to occupy small habitat patches is highlighted as conservational advantage that makes the possibility of implementation of various relatively simple conservation actions (Cane 2001). Our results allow us to propose several practices that should focus on ensuring floral diversity and pollinator nesting availabilities. Under windmills, pollinator food plants can be planted which should be native to this region and should be selected basing on the flowering period to provide food for pollinators during the entire season (as a guide, see Vaughan et al. 2007). Appropriate supervision should also prevent the area against overgrowth and maintaining an appropriate share of bare soil because such initial stages of vegetation succession usually have the highest wild plant species diversity (Tscharntke et al. 2011). Some studies have shown the positive effect of installing artificial nests on local populations of pollinators (Dicks et al. 2010). These actions may be introduced both at the stage of windmill construction and at already existing windmills. On the other hand, the negative effects related to service and maintaining of the windmills can also be taken into account. Activities such as the use of pesticides and intensive mowing and some earthworks or influx of pollutants from neighboring fields can harm pollinators and destroy their nests (Barmaz et al. 2012, Krupke et al. 2012). Thus, it is demanded to work out optimal management schemes that minimize the negative impact of these factors. For example, lower herbicide use near windmills could be advised. It is also important to raise public awareness of values of windmill sites, even if many of them may be perceived as ruderal and visually unattractive.

## Conclusion

In total, our study depicts the area of the wind farm as a potentially valuable habitat for wild plants and pollinating insects, organisms that decline in a farmland. However, we do not discuss studies that show negative effect of wind energy on other groups of animals, but we highlight that every enterprise may have also positive sides which may compensate or balance other threats. Thus, evaluation of effects of environmental changes on biodiversity should take into account different local processes. A lot of efforts are put into the creation of protected areas, while many anthropogenic and postindustrial sites conceal unexplored potential for sustaining biodiversity. This paper underlines the value of marginal, small habitats for biodiversity conservation that may alleviate negative effects of wind farm development and environmental conflicts in the anthropogenically modified landscapes.

## Electronic supplementary material

ESM 1 (DOCX 2845 kb)

## Footnotes

1 Map of renewable energy sources in Poland: http://www.ure.gov.pl/uremapoze/mapa.html (accessed 1 February 2017)

2 Wind farm developer website, nontechnical executive summary: http://www.edpr.com/sustainability/documents-library-and-publications/ (accessed 1 February 2017)
