# Supplementary material for: The association of windmills with conservation of pollinating insects and wild plants in homogeneous farmland of western Poland
Source: Environ Sci Pollut Res Int. 2017 Dec 15;25(7):6273–84. doi: 10.1007/s11356-017-0864-7 (PMC5846843; doi:10.1007/s11356-017-0864-7)
Supplement: Supplementary file 1 — (DOCX 2845 kb) [file 11356_2017_864_MOESM1_ESM.docx]

**The association of windmills with conservation of pollinating insects and wild plants in homogeneous farmland of western Poland**

Environmental Science and Pollution Research

Sylwia Pustkowiak^1*^, Weronika Banaszak – Cibicka, Łukasz Emil Mielczarek, Piotr Tryjanowski, Piotr Skórka

^1^Institute of Nature Conservation, Polish Academy of Sciences, Mickiewicza 33, 31-120, Kraków, Poland

* corresponding author e-mail: sylwia.pustkowiak@gmail.com

Supplementary Material 1


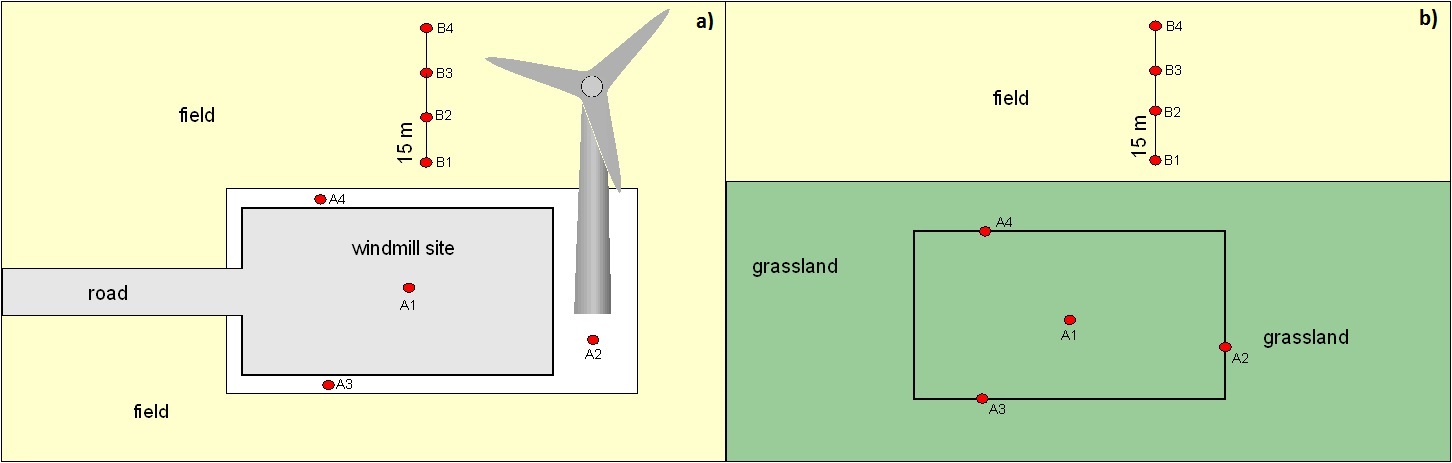


**Fig. S1** Distribution of sampling points within the windmill sites (a) and grasslands (b)


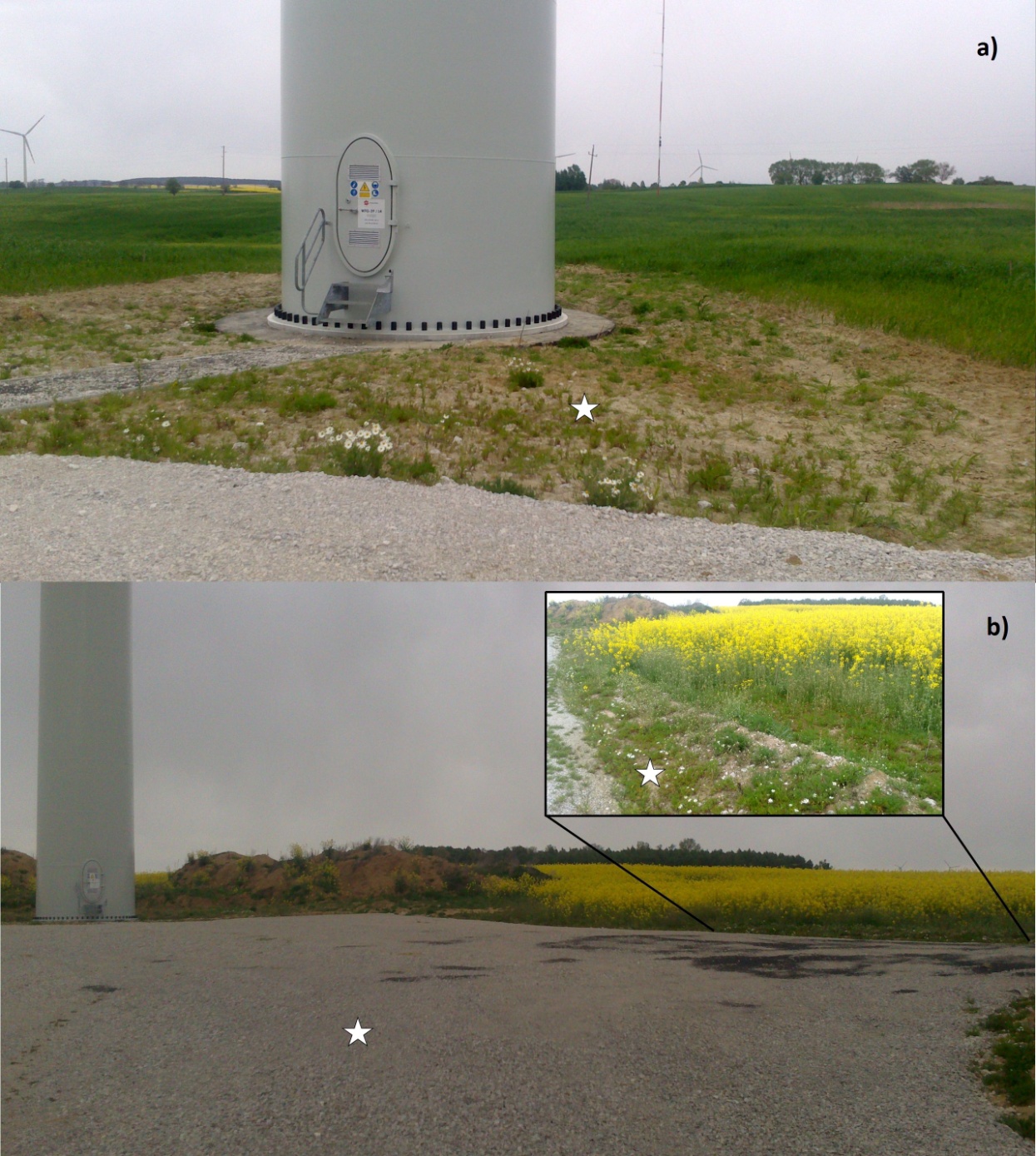


**Fig. S2** Examples of windmill plots within the studied wind farm in Gołańcz. White stars indicate location of sampling points in the vicinity of windmill


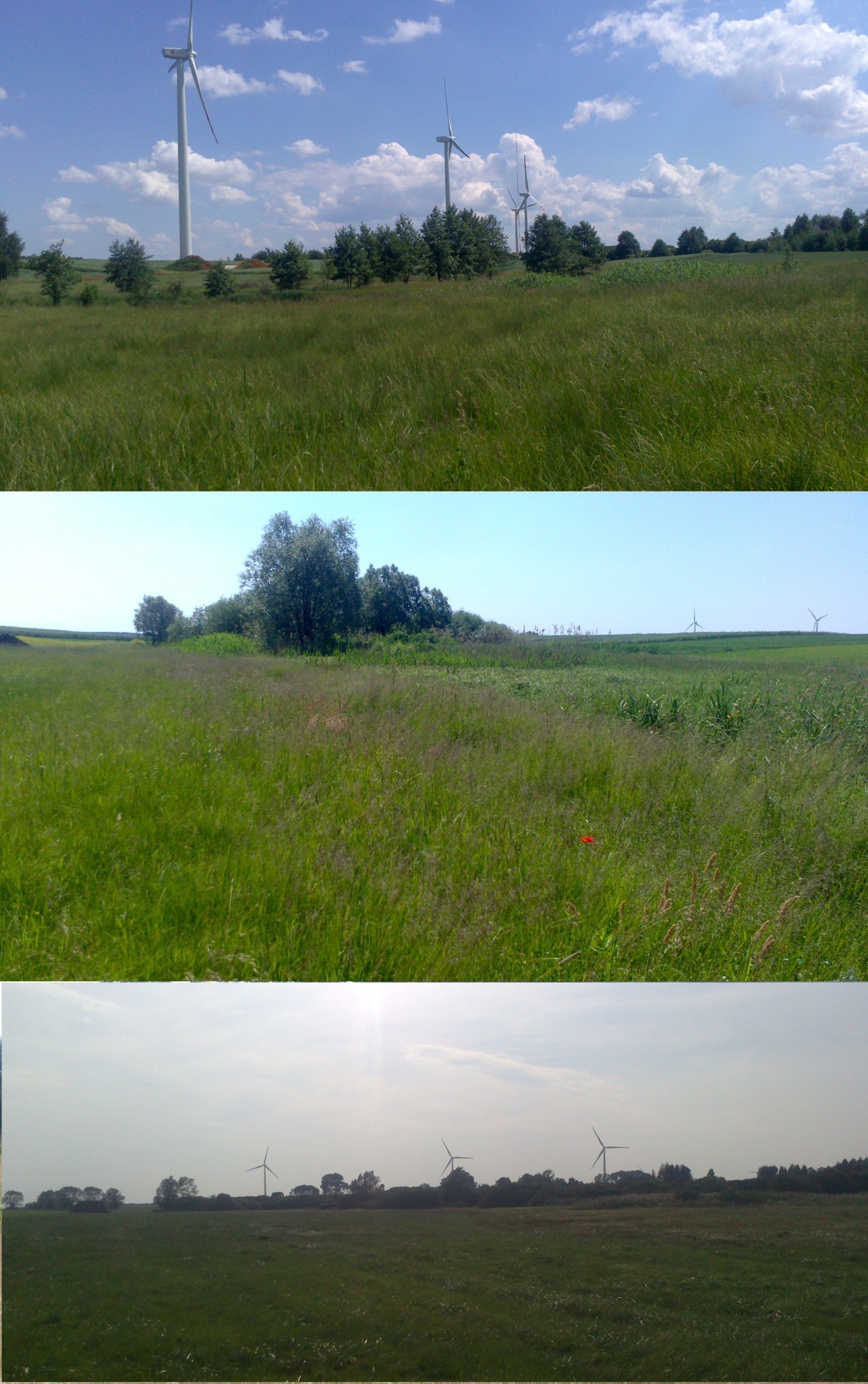


**Fig. S3** Examples of grassland patches within the studied wind farm in Gołańcz

**
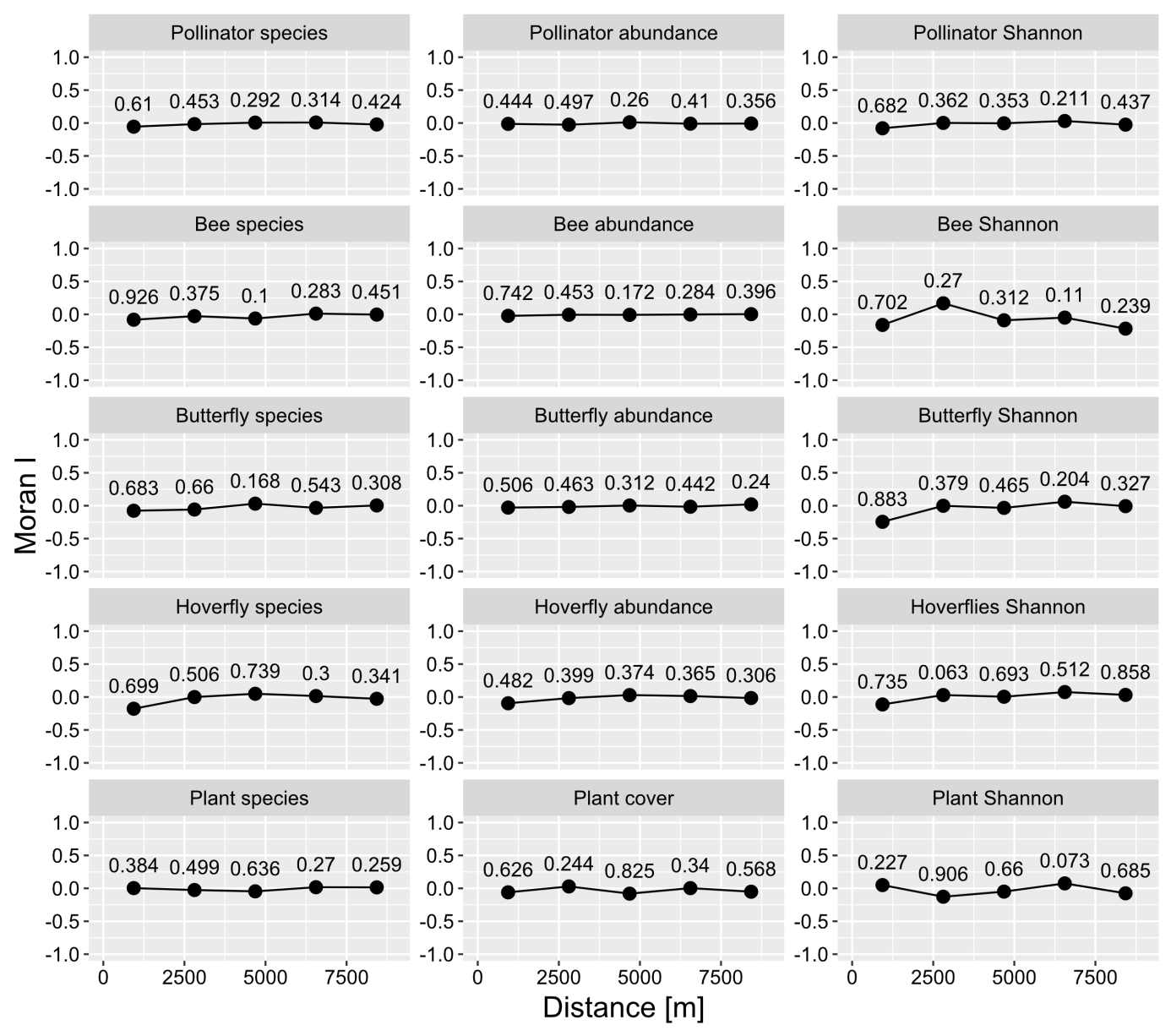
**

**Fig. S4** Spatial autocorrelograms with Moran *I* statistics for analyzed dependent variables. None estimate was statistically significant (P-values are inserted above dots representing distance classes)


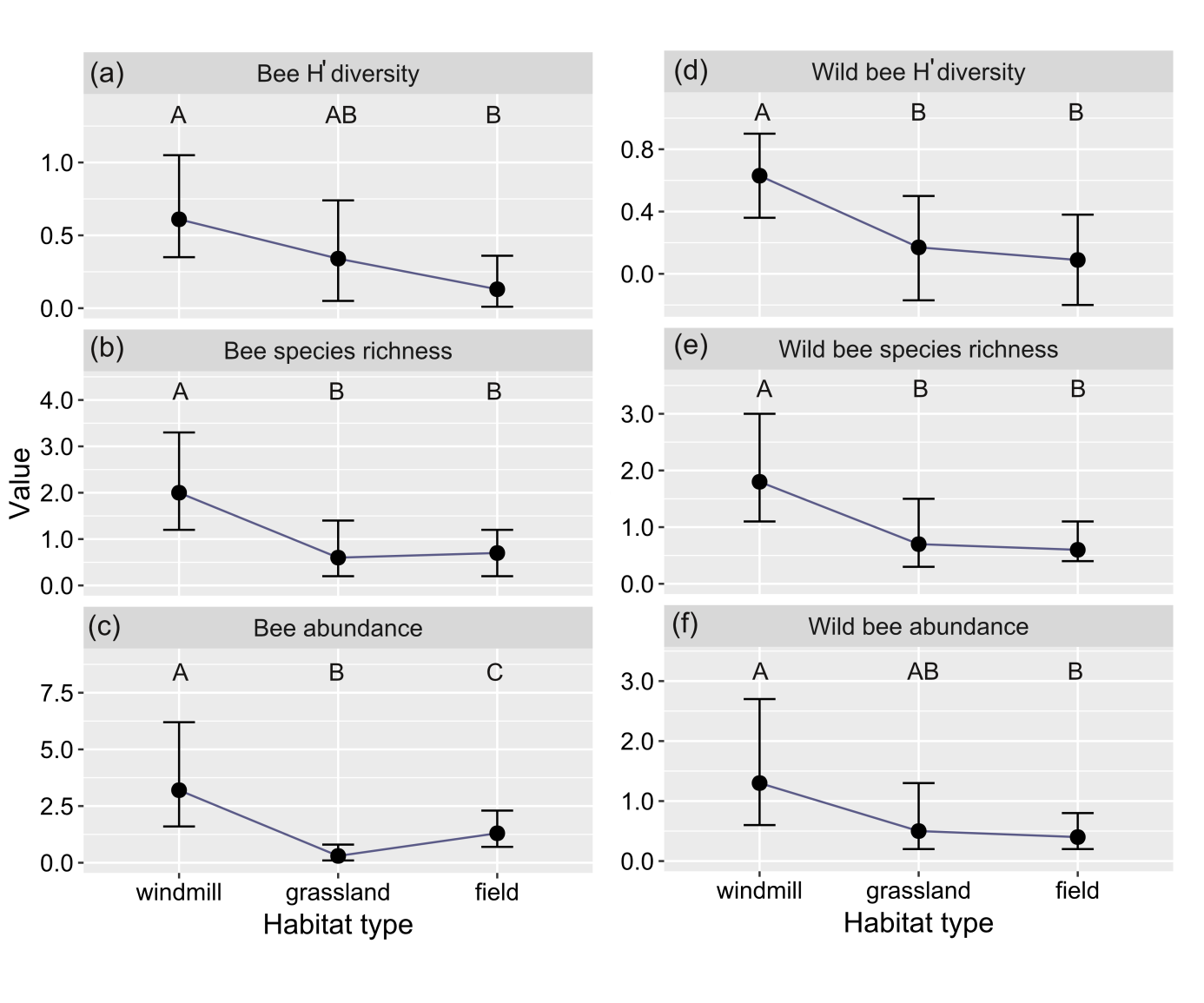


**Fig. S5** The relationship between habitat type and bee Shannon diversity H’ index (a), total number of bee species (b) and bee abundance within plots (c). Graphs d-f show the same estimations for wild bees (without *A. mellifera*). Points represent means estimated in generalized linear mixed models. Error bars show 0.95% confidence level also derived from generalized linear mixed models.


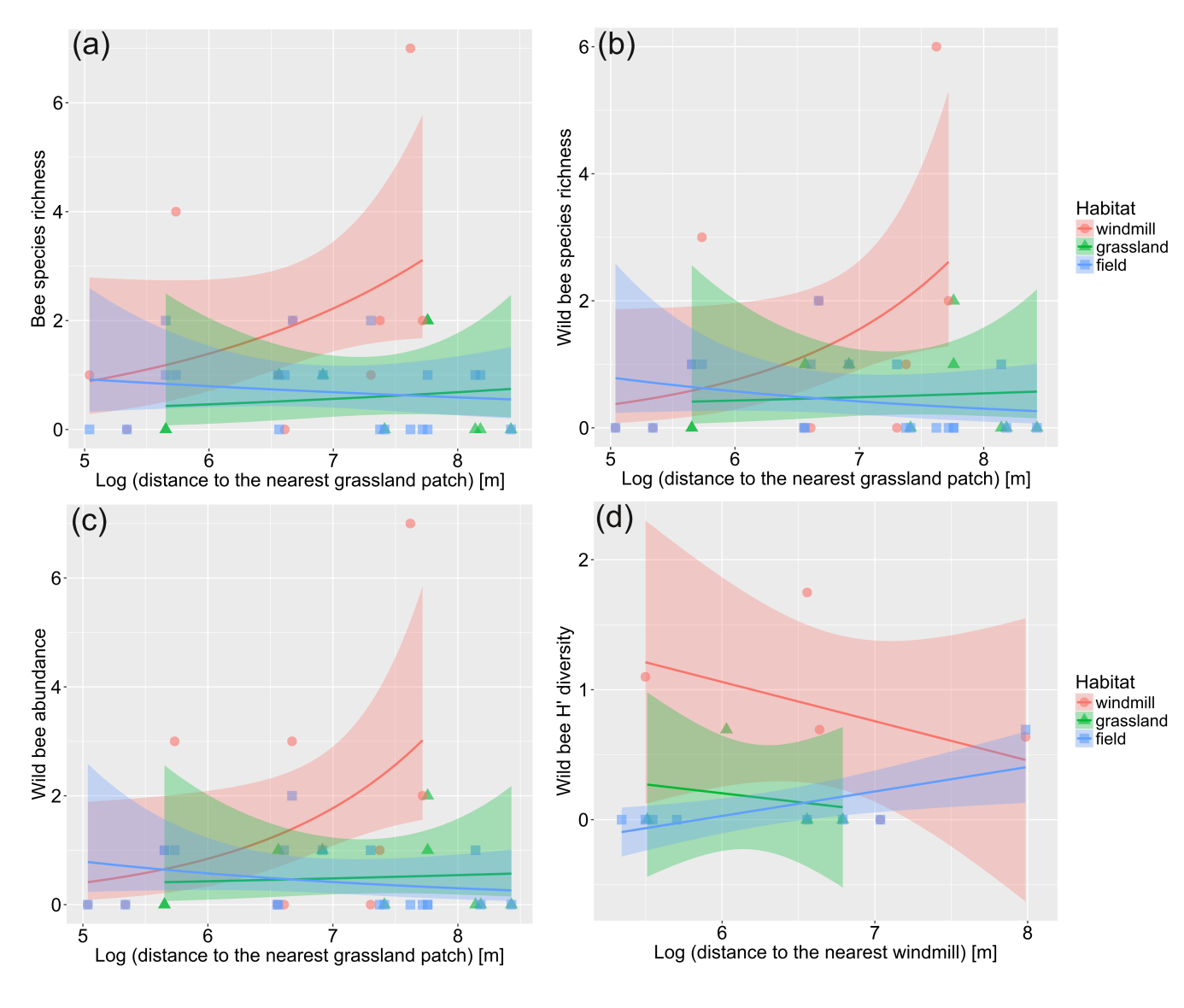
**Fig. S6** The effect of distance to the nearest grassland patch (a-c) and windmill (d) on bee species richness, wild bee species richness, wild bee abundance and wild bee diversity index. Shaded bands represents 95% confidence intervals


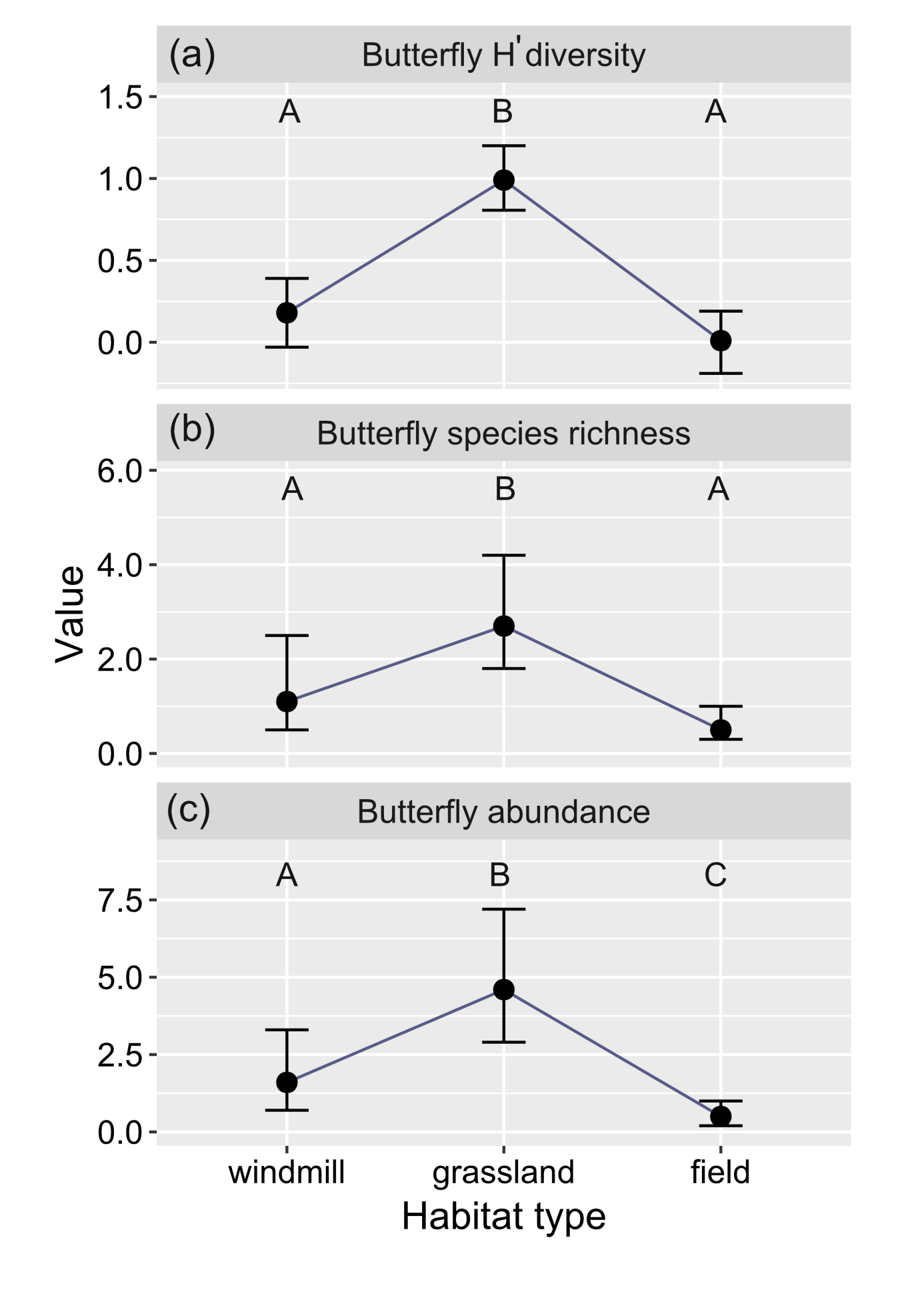


**Fig. S7** The relationship between habitat type and butterfly Shannon diversity H’ index (a), total number of butterfly species (b) and butterfly abundance (c). Further explanations: See: Fig. S5


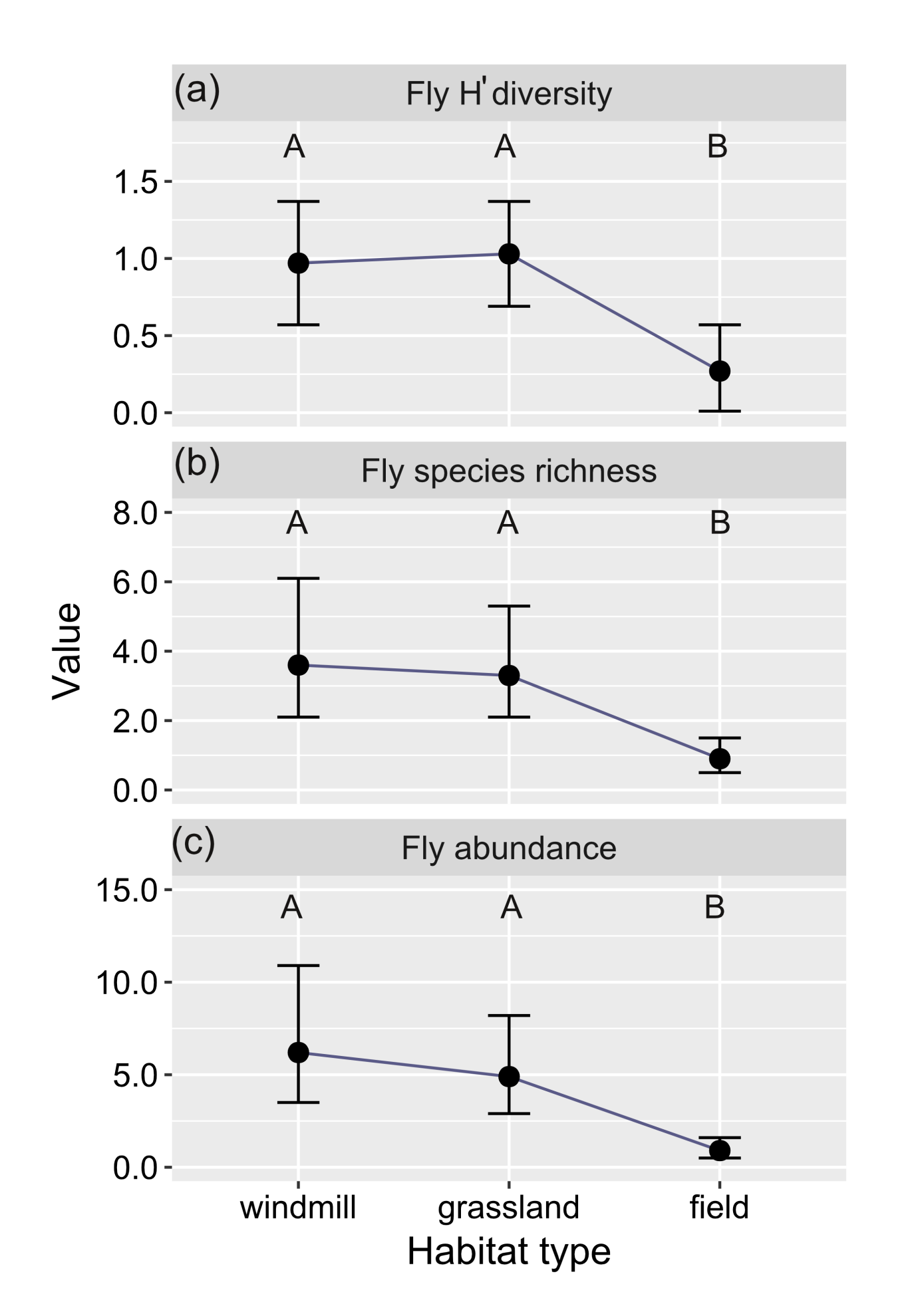


**Fig. S8** The relationship between habitat type and fly Shannon diversity H’ index (a), total number of fly species per plot (b) and fly abundance within plots (c). Further explanations: See: Fig. S5


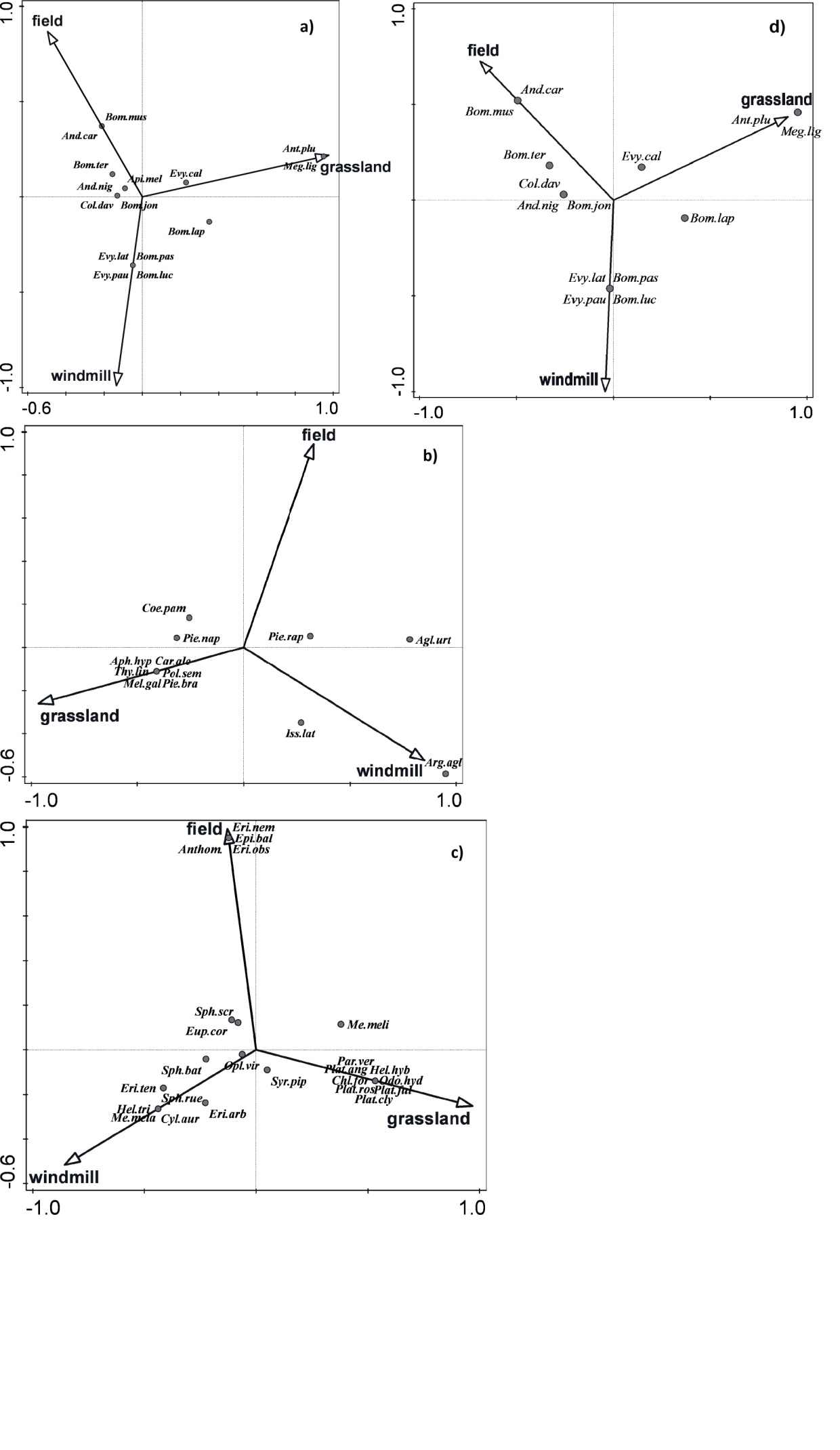


**Fig. S9** The CCA diagrams of relationships between habitats and (a) bees, (b) butterflies, (c) flies, d) wild bees along the first and second ordination axis

Table S1 The list of all captured species of pollinators

| No. | Species | Abbreviation | Order |
| --- | --- | --- | --- |
| 1 | *Colletes daviesanus* (Smith, 1846) | Col.dav | Hymenoptera |
| 2 | *Andrena nigroaenea* (Kirby, 1802) | And.nig | Hymenoptera |
| 3 | *Andrena carbonaria* (Linnaeus, 1767) | And.car | Hymenoptera |
| 4 | *Evylaeus calceatus* (Scopoli, 1763) | Evy.cal | Hymenoptera |
| 5 | *Evylaeus pauxillus* (Schenck, 1853) | Evy.pau | Hymenoptera |
| 6 | *Evylaeus laticeps* (Schenck, 1868) | Evy.lat | Hymenoptera |
| 7 | *Megachile ligniseca* (Kirby, 1802) | Meg.lig | Hymenoptera |
| 8 | *Apis mellifera* (Linnaeus, 1758) | Api.mel | Hymenoptera |
| 9 | *Bombus terrestris* (Linnaeus, 1758) | Bom.ter | Hymenoptera |
| 10 | *Bombus lapidarius* (Linnaeus, 1758) | Bom.lap | Hymenoptera |
| 11 | *Anthophora plumipes* (Pallas, 1772) | Ant.plu | Hymenoptera |
| 12 | *Bombus lucorum* (Linnaeus, 1761) | Bom.luc | Hymenoptera |
| 13 | *Bombus pascuorum* (Scopoli, 1763) | Bom.pas | Hymenoptera |
| 14 | *Bombus muscorum* (Linnaeus, 1758) | Bom.mus | Hymenoptera |
| 15 | *Bombus jonellus* (Kirby,1802) | Bom.jon | Hymenoptera |
| 16 | *Pieris rapae* (Linnaeus, 1758) | Pie.rap | Lepidoptera |
| 17 | *Pieris napi* (Linnaeus, 1758) | Pie.nap | Lepidoptera |
| 18 | *Pieris brassicae* (Linnaeus, 1758) | Pie.bra | Lepidoptera |
| 19 | *Aphantopus hyperantus* (Linnaeus, 1758) | Aph.hyp | Lepidoptera |
| 20 | *Aglais urticae* (Linnaeus, 1758) | Agl.urt | Lepidoptera |
| 21 | *Issoria lathonia* (Linnaeus, 1758) | Iss.lat | Lepidoptera |
| 22 | *Argynnis aglaja* (Linnaeus, 1758) | Arg.agl | Lepidoptera |
| 23 | *Coenonympha pamphilus* (Linnaeus, 1758) | Coe.pam | Lepidoptera |
| 24 | *Thymelicus lineola* (Ochsenheimer, 1808) | Thy.lin | Lepidoptera |
| 25 | *Carcharodus alceae* (Esper, 1780) | Car.alc | Lepidoptera |
| 26 | *Melanargia galathea* (Linnaeus, 1758) | Mel.gal | Lepidoptera |
| 27 | *Polyommatus semiargus* (Rottemburg, 1775) | Pol.sem | Lepidoptera |
| 28 | *Anthomyiidae sp.* | Anthom. | Diptera |
| 29 | *Chloromyia formosa* (Scopoli, 1763) | Chl.for | Diptera |
| 30 | *Cylindromyia auriceps* (Meigen, 1838) | Cyl.aur | Diptera |
| 31 | *Episyrphus balteatus* (De Geer, 1776) | Epi.bal | Diptera |
| 32 | *Eristalis arbustorum* (Linnaeus, 1758) | Eri.arb | Diptera |
| 33 | *Eristalis nemorum* (Linnaeus, 1758) | Eri.nem | Diptera |
| 34 | *Eristalis obscura* (Loew, 1886) | Eri.obs | Diptera |
| 35 | *Eristalis tenax* (Linnaeus, 1758) | Eri.ten | Diptera |
| 36 | *Eupeodes corollae* (Fabricius, 1794) | Eup.cor | Diptera |
| 37 | *Helophilus hybridus* (Loew, 1846) | Hel.hyb | Diptera |
| 38 | *Helophilus trivittatus* (Fabricius, 1805) | Hel.tri | Diptera |
| 39 | *Melanostoma cf. mellarium* (Meigen, 1822) | Me.mela | Diptera |
| 40 | *Melanostoma mellinum* (Linnaeus, 1758) | Me.meli | Diptera |
| 41 | *Odontomyia hydroleon* (Linnaeus 1758) | Odo.hyd | Diptera |
| 42 | *Oplodontha viridula* (Fabricius, 1775) | Opl.vir | Diptera |
| 43 | *Parhelophilus versicolor* (Fabricius, 1794) | Par.ver | Diptera |
| 44 | *Platycheirus angustatus* (Zetterstedt, 1843) | Plat.ang | Diptera |
| 45 | *Platycheirus clypeatus* (Meigen, 1822) | Plat.cly | Diptera |
| 46 | *Platycheirus fulviventris* (Macquart, 1829) | Plat.ful | Diptera |
| 47 | *Platycheirus rosarum* (Fabricius, 1787) | Plat.ros | Diptera |
| 48 | *Sphaerophoria batava* (Goeldlin, 1974) | Sph.bat | Diptera |
| 49 | *Sphaerophoria rueppelli* (Wiedemann, 1830) | Sph.rue | Diptera |
| 50 | *Sphaerophoria scripta* (Linnaeus, 1758) | Sph.scr | Diptera |
| 51 | *Syritta pipiens* (Linnaeus, 1758) | Syr.pip | Diptera |

Table S2 The list of all plant species

| No. | Species | Abbreviation |
| --- | --- | --- |
| 1 | *Achillea millefolium* (Linnaeus, 1753) | Ach.mil |
| 2 | *Agropyron repens* (Linnaeus) Gould | Agr.rep |
| 3 | *Alopecurus myosuroides* (Huds., 1762) | Alo.myo |
| 4 | *Alopecurus pratensis* (Linnaeus, 1753) | Alo.pra |
| 5 | *Amaranthus hybridus* (Linnaeus, 1753) | Ama.hyb |
| 6 | *Amaranthus retroflexus* (Linnaeus, 1753) | Ama.ret |
| 7 | *Anagallis arvensis* (Linnaeus, 1753) | Ana.arv |
| 8 | *Anchusa arvensis* (Linnaeus) M. Bieb., 1808 | Anc.arv |
| 9 | *Apera spica-venti* (Linnaeus) P.Beauv, 1812 | Ape.spi |
| 10 | *Arctium tomentosum* (MilLinnaeus, 1768) | Arc.tom |
| 11 | *Armoracia rusticana* (G. Gaertn. et aLinnaeus., 1800) | Arm.rus |
| 12 | *Arrhenatherum elatius* (Linnaeus) P. Beauv. ex J. & C. Presl, 1819 | Arr.ela |
| 13 | *Artemisia absinthium* (Linnaeus, 1753) | Art..abs |
| 14 | *Artemisia campestris* (Linnaeus, 1753) | Art..cam |
| 15 | *Artemisia vulgaris* (Linnaeus, 1753) | Art..vul |
| 16 | *Astragalus glycyphyllos* (Linnaeus, 1753) | Ast.gly |
| 17 | *Avena fatua* (Linnaeus, 1753) | Ave.fat |
| 18 | *Avena sativa* (Linnaeus) | Ave.sat |
| 19 | *Berteroa incana* (Linnaeus) DC., 1821 | Ber.inc |
| 20 | *Brassica napus* (Linnaeus, 1753) | Bra.nap |
| 21 | *Bromus hordeaceus* (Linnaeus, 1753) | Bro.hor |
| 22 | *Bromus racemosus* (Linnaeus) | Bro.rac |
| 23 | *Bromus secalinus* (Linnaeus, 1753) | Bro.sec |
| 24 | *Calamagrostis epigejos* (Linnaeus) Roth, 1788 | Cal.epi |
| 25 | *Capsella bursa pastoris* (Linnaeus) Medik., 1792 | Cap.bur |
| 26 | *Carduus acanthoides* (Linnaeus, 1753) | Car.aca |
| 27 | *Carduus crispus* (Linnaeus, 1753) | Car.cri |
| 28 | *Carex acutiformis* (Linnaeus) | Car.acu |
| 29 | *Carex flava* (Linnaeus, 1753) | Car.fla |
| 30 | *Carex panicea* (Linnaeus, 1753) | Car.pan |
| 31 | *Centaurea cyanus* (Linnaeus, 1753) | Cen.cya |
| 32 | *Centaurea jacea* (Linnaeus, 1753) | Cen.jac |
| 33 | *Chenopodium album* (Linnaeus, 1753) | Che.alb |
| 34 | *Cichorium intybus* (Linnaeus, 1753) | Cic.int |
| 35 | *Cirsium acaule* (Linnaeus) Scop., 1769 | Cir.aca |
| 36 | *Cirsium arvense* (Linnaeus) Scop., 1772 | Cir.arv |
| 37 | *Consolida regalis* (S.F. Gray, 1821) | Con.reg |
| 38 | *Convolvulus arvensis* (Linnaeus, 1753) | Con.arv |
| 39 | *Conyza canadensis* (Linnaeus, 1753) | Con.can |
| 40 | *Dactylis glomerata* (Linnaeus, 1753) | Dac.glo |
| 41 | *Daucus carota* (Linnaeus, 1753) | Dau.car |
| 42 | *Deschampsia caespitosa* (Linnaeus) P.B. | Des.cae |
| 43 | *Deschampsia flexuosa* (Linnaeus) | Des.fle |
| 44 | *Dipsacus sylvestris* (Huds.) | Dip.syl |
| 45 | *Echinochloa crus-galli* (Linnaeus) P.Beauv., 18,12 | Ech.cru |
| 46 | *Epilobium hirsutum* (Linnaeus) | Epi.hir |
| 47 | *Equisetum arvense* (Linnaeus) | Equ.arv |
| 48 | *Euphorbia helioscopia* (Linnaeus, 1753) | Eup.hel |
| 49 | Fagopyrum tataricum (Linnaeus) Gaertn., 1790 | Fag.tat |
| 50 | *Festuca pratensis* (Huds.) | Fes.pra |
| 51 | *Festuca rubra* (Linnaeus, 1753) | Fes.rub |
| 52 | *Galeopsis tetrahit* (Linnaeus, 1753) | Gal.tet |
| 53 | *Galinsoga parviflora* (Cav., 1796) | Gal.par |
| 54 | *Galium mollugo* (Linnaeus, 1753) | Gal.mol |
| 55 | *Galium uliginosum* (Linnaeus, 1753) | Gal.uli |
| 56 | *Geranium pusillum* (Linnaeus, 1759) | Ger.pus |
| 57 | *Glechoma hederacea* (Linnaeus, 1753) | Gle.hed |
| 58 | *Hordeum vulgare* (Linnaeus, 1753) | Hor.vul |
| 59 | *Hypericum perforatum* (Linnaeus, 1753) | Hyp.per |
| 60 | *Hypochoeris radicata* (Linnaeus, 1753) | Hyp.rad |
| 61 | *Iris sibirica* (Linnaeus, 1753) | Iri.sib |
| 62 | *Juncus bufonius* (Linnaeus, 1753) | Jun.buf |
| 63 | *Juncus effusus* (Linnaeus, 1753) | Jun.eff |
| 64 | *Knautia arvensis* (Linnaeus) Coult. | Kna.arv |
| 65 | *Lamium purpureum* (Linnaeus, 1753) | Lam.pur |
| 66 | *Leontodon autumnalis* (Linnaeus, 1753) | Leo.aut |
| 67 | *Leontodon hispidus* (Linnaeus, 1753) | Leo.his |
| 68 | *Lepiduim campestre* (Linnaeus) W. T. Aiton, 1812 | Lep.cam |
| 69 | *Lepidum ruderale* (Linnaeus, 1753) | Lep.rud |
| 70 | *Lolium multiflorum* (Lam.) | Lol.mul |
| 71 | *Lolium perenne* (Linnaeus) | Lol.per |
| 72 | *Lotus corniculatus* (Linnaeus, 1753) | Lot.cor |
| 73 | *Luzula campestris* (Linnaeus) DC., 1805 | Luz.cam |
| 74 | *Lycopus europaeus* (Linnaeus, 1753) | Lyc.eur |
| 75 | *Lythrum salicaria* (Linnaeus, 1753) | Lyt.sal |
| 76 | *Matricaria chamomilla* (Linnaeus, 1753) | Mat.cha |
| 77 | *Matricaria discoidea* (DC., 1838) | Mat.dis |
| 78 | *Medicago falcata* (Linnaeus, 1753) | Med.fal |
| 79 | *Melandrium album* (Garcke, 1858) | Mel.alb |
| 80 | *Melilotus albus* (Medik., 1787) | Mel.alb |
| 81 | *Mentha aquatica* (Linnaeus, 1753) | Men.aqu |
| 82 | *Mentha arvensis* (Linnaeus, 1753) | Men.arv |
| 83 | *Myosotis arvensis* (Linnaeus) Hill, 1764 | Myo.arv |
| 84 | *Papaver dubium* (Linnaeus, 1753) | Pap.dub |
| 85 | *Pastinaca sativa* (Linnaeus, 1753) | Pas.sat |
| 86 | *Phragmites australis* (Cav.)Trin. ex Steud, 1841 | Phr.aus |
| 87 | *Picris hieracioides* (Linnaeus, 1753) | Pic.hie |
| 88 | *Pimpinella major* (Linnaeus) Huds., 1762 | Pim.maj |
| 89 | *Pinus sylvestris* (Linnaeus, 1753) | Pin.syl |
| 90 | *Plantago lanceolata* (Linnaeus, 1753) | Pla.lan |
| 91 | *Plantago major* (Linnaeus, 1753) | Pla.maj |
| 92 | *Poa annua* (Linnaeus) | Poa.ann |
| 93 | *Poa pratensis* (Linnaeus) | Poa.pra |
| 94 | *Polygonum amphibium* (Linnaeus) Delarbre, 1800 | Pol.amp |
| 95 | *Polygonum aviculare* (Linnaeus, 1753) | Pol.avi |
| 96 | *Polygonum persicaria* (Linnaeus, 1753) | Pol.per |
| 97 | *Potentilla anserina* (Linnaeus, 1753) | Pot.ans |
| 98 | *Potentilla argentea* (Linnaeus, 1753) | Pot.arg |
| 99 | *Potentilla reptans* (Linnaeus, 1753) | Pot.rep |
| 100 | *Ranunculus acris* (Linnaeus, 1753) | Ran.acr |
| 101 | *Ranunculus repens* (Linnaeus, 1753) | Ran.rep |
| 102 | *Rubus Sp.* (Linnaeus, 1753) | Rub.Sp |
| 103 | *Rumex acetosa* (Linnaeus, 1753) | Rum.ace |
| 104 | *Rumex acetosella* (Linnaeus, 1753) | Rum.ace |
| 105 | *Rumex crispus* (Linnaeus, 1753) | Rum.cri |
| 106 | *Sambucus nigra* (Linnaeus, 1753) | Sam.nig |
| 107 | *Sambucus racemosa* (Linnaeus, 1753) | Sam.rac |
| 108 | *Secale cereale* (Linnaeus, 1753) | Sec.cer |
| 109 | *Senecio jacobaea* (Gaertn., 1791) | Sen.jac |
| 110 | *Senecio vulgaris* (Linnaeus, 1753) | Sen.vul |
| 111 | *Sinapis arvensis* (Linnaeus, 1753) | Sin.arv |
| 112 | *Solanum dulcamara* (Linnaeus, 1753) | Sol.dul |
| 113 | *Solidago virgaurea* (Linnaeus, 1753) | Sol.vir |
| 114 | *Sonchus arvensis* (Linnaeus, 1753) | Son.arv |
| 115 | *Sorbus aucuparia* (Linnaeus, 1753) | Sor.auc |
| 116 | *Spergula arvensis* (Linnaeus, 1753) | Spe.arv |
| 117 | *Stellaria media* (Linnaeus) VilLinnaeus, 1784 | Stel.med |
| 118 | *Tanacetum vulgare* (Linnaeus, 1753) | Tan.vul |
| 119 | *Taraxacum officinale* (F.H. Wigg., 1780) | Tar.off |
| 120 | *Taraxacum palustre* (Lyons) Symons | Tar.pal |
| 121 | *Thlaspi arvense* (Linnaeus, 1753) | Thl.arv |
| 122 | *Tragopogon pratensis* (Linnaeus, 1753) | Tra.pra |
| 123 | *Trifolium dubium* (Sibth., 1794) | Tri.dub |
| 124 | *Trifolium medium* (Linnaeus, 1759) | Tri.med |
| 125 | *Trifolium pratense* (Linnaeus, 1753) | Tri.pra |
| 126 | *Trifolium repens* (Linnaeus, 1753) | Tri.rep |
| 127 | *Tripleurospermum inodorum* (Linnaeus) | Tri.ino |
| 128 | *Triticum aestivum* (Linnaeus, 1753) | Tri.aes |
| 129 | *Tussilago farfara* (Linnaeus, 1753) | Tus.far |
| 130 | *Typha angustifolia* (Linnaeus, 1753) | Typ.ang |
| 131 | *Urtica dioica* (Linnaeus, 1753) | Urt.dio |
| 132 | *Vicia cracca* (Linnaeus, 1753) | Vic.cra |
| 133 | *Vicia sativa* (Linnaeus, 1753) | Vic.sat |
| 134 | *Viola arvensis* (Murr., 1770) | Vio.arv |

Table S3 Summary of generalized linear mixed models explaining diversity, species richness and abundance of bees (with and without honeybee), butterflies and flies in the three studied habitats. Windmill site is used as a reference level of habitat. Plot ID is used as a random factor. Explanations: DistanceW – distance to the nearest windmill, DistanceG – distance to the nearest grassland. Effect of interaction term between Habitat and DistanceW and/or Habitat and DistanceG are included in presented model only if it was significant. Statistically significant effects are marked with bold

| Explanatory variables | Estimate | Std.Error | t/z - value | P - value |
| --- | --- | --- | --- | --- |
| GLMM7 (bee H’ diversity index) |  |  |  |  |
| Intercept | 0.60 | 0.15 | 3.97 | **0.001** |
| Habitat=grassland | -0.26 | 0.26 | -0.97 | 0.342 |
| Habitat=field | -0.47 | 0.20 | -2.36 | **0.028** |
| DistanceW | -0.19 | 0.16 | -1.75 | 0.255 |
| DistanceG | 0.28 | 0.19 | 1.46 | 0.162 |
| Habitat=windmill | 0* |  |  |  |
| GLMM8 (bee species richness) |  |  |  |  |
| Intercept | 0.65 | 0.26 | 2.55 | **0.011** |
| Habitat=grassland | -1.20 | 0.48 | -2.49 | **0.013** |
| Habitat=field | -1.05 | 0.35 | -2.97 | **0.003** |
| DistanceW | -0.46 | 0.35 | -1.32 | 0.185 |
| DistanceG | 0.67 | 0.31 | 2.17 | **0.030** |
| Habitat=grassland*DistanceG | -0.51 | 0.59 | -0.87 | 0.386 |
| Habitat=field*DistanceG | -0.97 | 0.43 | -2.22 | **0.026** |
| Habitat=windmill | 0* |  |  |  |
| GLMM9 (bee abundance) |  |  |  |  |
| Intercept | 1.02 | 0.34 | 3.01 | **0.003** |
| Habitat=grassland | -2.40 | 0.54 | -4.45 | **<0.001** |
| Habitat=field | -0.94 | 0.32 | -2.95 | **0.003** |
| DistanceW | 0.13 | 0.31 | 0.44 | 0.664 |
| DistanceG | 0.10 | 0.32 | 0.31 | 0.753 |
| Habitat=windmill | 0* |  |  |  |
| GLMM10 (wild bee H’ diversity index) |  |  |  |  |
| Intercept | 0.84 | 0.19 | 4.47 | **0.001** |
| Habitat=grassland | -0.66 | 0.28 | -2.36 | **0.033** |
| Habitat=field | -0.75 | 0.24 | -3.15 | **0.007** |
| DistanceW | -0.30 | 0.16 | -1.95 | 0.075 |
| DistanceG | 0.07 | 0.15 | 0.49 | 0.631 |
| Habitat=grassland*DistanceW | 0.28 | 0.30 | 0.92 | 0.373 |
| Habitat=field*DistanceW | 0.42 | 0.02 | 21.0 | **<0.001** |
| Habitat=windmill | 0* |  |  |  |
| GLMM11 (wild bee species richness) |  |  |  |  |
| Intercept | 0.15 | 0.36 | 0.42 | 0.672 |
| Habitat=grassland | -0.97 | 0.58 | -1.67 | 0.095 |
| Habitat=field | -1.24 | 0.47 | -2.62 | **0.009** |
| DistanceW | -0.05 | 0.27 | -0.19 | 0.847 |
| DistanceG | 0.73 | 0.42 | 1.69 | 0.090 |
| Habitat=grassland*DistanceG | -0.56 | 0.66 | -0.84 | 0.402 |
| Habitat=field*DistanceG | -1.04 | 0.53 | -1.97 | **0.048** |
| Habitat=windmill | 0* |  |  |  |
| GLMM12 (wild bee abundance) |  |  |  |  |
| Intercept | 0.21 | 0.37 | 0.56 | 0.575 |
| Habitat=grassland | -1.07 | 0.59 | -1.81 | 0.071 |
| Habitat=field | -1.22 | 0.44 | -2.78 | **0.005** |
| DistanceW | 0.05 | 0.42 | 0.12 | 0.903 |
| DistanceG | 1.07 | 0.58 | 1.84 | 0.066 |
| Habitat=grassland*DistanceG | -1.30 | 0.73 | -1.76 | 0.079 |
| Habitat=field*DistanceG | -1.67 | 0.71 | -2.34 | **0.019** |
| Habitat=windmill | 0* |  |  |  |
| GLMM13 (butterfly H’ diversity index) |  |  |  |  |
| Intercept | 0.11 | 0.10 | 1.11 | 0.279 |
| Habitat=grassland | 0.90 | 0.12 | 7.25 | **<0.001** |
| Habitat=field | -0.05 | 0.11 | -0.48 | 0.642 |
| DistanceW | -0.01 | 0.10 | -0.02 | 0.996 |
| DistanceG | 0.05 | 0.07 | 0.66 | 0.552 |
| Habitat=windmill | 0* |  |  |  |
| GLMM14 (butterfly species richness) |  |  |  |  |
| Intercept | <0.001 | 0.32 | <0.001 | 1.000 |
| Habitat=grassland | 1.10 | 0.37 | 3.01 | **0.003** |
| Habitat=field | -0.92 | 0.47 | -1.93 | 0.053 |
| DistanceW | -0.36 | 0.20 | -1.81 | 0.071 |
| DistanceG | 0.13 | 0.17 | 0.783 | 0.434 |
| Habitat=windmill | 0* |  |  |  |
| GLMM15 (butterfly abundance) |  |  |  |  |
| Intercept | 0.40 | 0.32 | 1.23 | 0.218 |
| Habitat=grassland | 1.13 | 0.39 | 2.95 | **0.003** |
| Habitat=field | -1.18 | 0.41 | -2.87 | **0.004** |
| DistanceW | -0.54 | 0.25 | -2.10 | **0.036** |
| DistanceG | 0.28 | 0.21 | 1.32 | 0.185 |
| Habitat=windmill | 0* |  |  |  |
| GLMM16 (fly H’ diversity index) |  |  |  |  |
| Intercept | 1.03 | 0.17 | 5.98 | **<0.001** |
| Habitat=grassland | -0.09 | 0.22 | -0.41 | 0.687 |
| Habitat=field | -0.73 | 0.18 | -3.93 | **0.002** |
| DistanceW | -0.30 | 0.14 | -2.12 | 0.054 |
| DistanceG | 0.20 | 0.15 | 1.26 | 0.223 |
| Habitat=windmill | 0* |  |  |  |
| GLMM17 (fly species richness) |  |  |  |  |
| Intercept | 1.10 | 0.22 | 4.96 | **<0.001** |
| Habitat=grassland | 0.08 | 0.30 | 0.27 | 0.787 |
| Habitat=field | -1.29 | 0.31 | -4.22 | **<0.001** |
| DistanceW | -0.11 | 0.17 | -0.63 | 0.528 |
| DistanceG | 0.12 | 0.16 | 0.72 | 0.474 |
| Habitat=windmill | 0* |  |  |  |
| GLMM18 (fly abundance) |  |  |  |  |
| Intercept | 1.67 | 0.22 | 7.67 | **<0.001** |
| Habitat=grassland | -0.12 | 0.29 | -0.40 | 0.689 |
| Habitat=field | -1.76 | 0.27 | -6.54 | **<0.001** |
| DistanceW | -0.83 | 0.68 | -1.21 | 0.242 |
| DistanceG | 0460 | 0.69 | 0.66 | 0.515 |
| Habitat=windmill | 0* |  |  |  |

* a reference category

Table S4 The detailed results of CCA analysis (with habitat type as explanatory variable and plot ID as supplementary variable) on particular groups of pollinators. Term Effect shows the significance of each habitat type. P-value is adjusted by Bonferroni correction. Statistically significant effect are marked with bold

|  |  | Term Effects: | | | |
| --- | --- | --- | --- | --- | --- |
| Group | Habitat | Explains % | pseudo-F | P | P(adj) |
| bees | grassland | 6.8 | 1.5 | 0.132 | 0.40 |
|  | field | 4.2 | 0.9 | 0.590 | 1.00 |
|  | square | 3.3 | 0.7 | 0.806 | 1.00 |
|  | grassland | 7.30 | 1.20 | 0.25 | 0.738 |
| wild bees | field | 6.50 | 1.00 | 0.35 | 1.000 |
|  | square | 5.30 | 0.80 | 0.68 | 1.000 |
| butterflies | **grassland** | **10.9** | **2.8** | **0.004** | **0.01** |
|  | square | 8.7 | 2.2 | 0.038 | 0.11 |
|  | field | 2.3 | 0.5 | 0.874 | 1.00 |
| flies | **grassland** | **7.1** | **2.2** | **0.002** | **0.006** |
|  | **square** | **6.4** | **1.9** | **0.004** | **0.012** |
|  | field | 3.9 | 1.1 | 0.192 | 0.576 |
